# Proteins Involved in Endothelial Function and Inflammation Are Implicated in Cerebral Small Vessel Disease

**DOI:** 10.1161/STROKEAHA.124.049079

**Published:** 2025-02-05

**Authors:** Zihan Sun, Eric L. Harshfield, Frank-Erik de Leeuw, Stephen Burgess, Adam S. Butterworth, Niels P. Riksen, Ziad Mallat, Hugh S. Markus

**Affiliations:** Stroke Research Group, Department of Clinical Neurosciences (Z.S., E.L.H., H.S.M.), University of Cambridge, United Kingdom.; British Heart Foundation Cardiovascular Epidemiology Unit, Department of Public Health and Primary Care (S.B., A.S.B.), University of Cambridge, United Kingdom.; British Heart Foundation Centre of Research Excellence (S.B., A.S.B.), University of Cambridge, United Kingdom.; Medical Research Council Biostatistics Unit, Cambridge Institute of Public Health (S.B.), University of Cambridge, United Kingdom.; National Institute for Health and Care Research Blood and Transplant Research Unit in Donor Health and Genomics (A.S.B.), University of Cambridge, United Kingdom.; The Victor Phillip Dahdaleh Heart and Lung Research Institute, Section of Cardiorespiratory Medicine, Department of Medicine (Z.M.), University of Cambridge, United Kingdom.; Department of Neurology (F.-E.d.L.), Radboud University Medical Centre, Nijmegen, the Netherlands.; Department of Internal Medicine (N.P.R.), Radboud University Medical Centre, Nijmegen, the Netherlands.; Donders Institute for Brain, Cognition, and Behaviour, Radboud University, Nijmegen, the Netherlands (F.-E.d.L.).; Health Data Research UK Cambridge, Wellcome Genome Campus and University of Cambridge, United Kingdom (A.S.B.).; Université de Paris, Inserm U970, Paris Cardiovascular Research Centre, France (Z.M.).

**Keywords:** cerebral small vessel diseases, dementia, inflammation, Mendelian randomization analysis, stroke

## Abstract

**BACKGROUND:**

Endothelial dysfunction and inflammation have been implicated in the pathophysiology of cerebral small vessel disease (SVD). However, whether they are causal, and if so which components of the pathways represent potential treatment targets, remains uncertain.

**METHODS:**

Two-sample Mendelian randomization (MR) was used to test the association between the circulating abundance of 996 proteins involved in endothelial dysfunction and inflammation and SVD. The genetic instruments predicting protein levels were obtained from the Iceland 36K (n=35 892) and the UK Biobank Proteomics (n=34 557) cohorts, both of which were longitudinal studies with follow-up from 2000 to 2023 and 2006 to 2023, respectively. SVD was represented by lacunar stroke (n=6030 cases) and 5 neuroimaging features (white matter hyperintensities [n=55 291], diffusion tensor imaging metrics: mean diffusivity [n=36 460] and fractional anisotropy [n=36 533], extensive white matter perivascular space burden [n=9324 cases], and cerebral microbleeds [n=3556 cases]). Among the proteins supported by causal evidence from the MR, cross-sectional analysis was performed to assess their associations with cognitive performance; survival analysis with Fine-Gray models was applied to examine their associations with incident all-cause dementia and stroke within the UK Biobank Proteomics cohort.

**RESULTS:**

MR suggested COL2A1 (collagen type II α-1 chain) was associated with lacunar stroke (odds ratio, 0.89 [95% CI, 0.86–0.91]; *P*=5×10^−5^). Moreover, 12 proteins related to endothelial function and inflammation were associated with neuroimaging features of SVD. Cross-sectional analyses showed 5 of the 13 proteins (EPHA2 [ephrin type-A receptor 2], METAP1D [methionine aminopeptidase 1D, mitochondrial], FLT4 [vascular endothelial growth factor receptor 3], COL2A1, and TIMD4 [T-cell immunoglobulin and mucin domain–containing protein 4]) were associated with cognitive performance with effects concordant with their MR findings. Survival analyses with the Fine-Gray models indicated that 5 of the 13 proteins (EPHA2, METAP1D, FLT4, APOE [apolipoprotein E], and PDE5A [cGMP-specific 3',5'-cyclic phosphodiesterase]) were associated with the risk of all-cause dementia or stroke independent of age and sex, consistent with their MR evidence.

**CONCLUSIONS:**

Our findings suggest that endothelial-platelet activation and complement-mediated regulation of inflammation play roles in SVD and identify potential therapeutic targets and pathways.

Cerebral small vessel disease (SVD) causes lacunar stroke (LS) and intracerebral hemorrhage and is the most common vascular contributor to dementia.^1^ SVD encompasses a range of pathologies that occur in the small arteries, arterioles, venules, and capillaries of the brain. These pathological changes lead to characteristic changes on magnetic resonance imaging (MRI), namely lacunar (small deep) infarcts, white matter hyperintensities (WMH), cerebral microbleeds (CMB), and enlarged perivascular spaces (EPVS).^2^ These pathological changes become highly prevalent with age, are associated with cognitive decline, and predict future stroke and dementia.^3^

Although the mechanisms underlying SVD remain partially understood, endothelial dysfunction and inflammation have been proposed as important contributors.^4–6^ Prior studies have suggested that endothelial dysfunction is an early step in SVD pathogenesis.^4,7^ In response to perturbations, endothelial cells could transition into an active proinflammatory state which expresses adhesion molecules to recruit circulating leukocytes.^7^ This proinflammatory phenotype also triggers microglial activation, cytokine production, macrophage infiltration, oxidative stress, and cell apoptosis.^6^ If left untampered, these responses would in turn exacerbate endothelial dysfunction, cause blood-brain barrier breakdown, and eventually result in parenchymal brain damage.^6^

Although the involvement of multiple pathogenic pathways of SVD makes it particularly hard to pinpoint causal biomarkers and identify therapeutic targets, recent advancement in omics technologies and blood-based biomarker assessment offers a window of opportunity. Proteins are the major signaling transducers and effectors in the pathways of endothelial function and inflammation. To date, high-throughput assays have been developed to allow the measurement of thousands of proteins in population-based studies. With these data, genome-wide association studies (GWAS) can reveal protein quantitative trait loci (pQTLs) that regulate plasma protein abundance.^8,9^ Mendelian randomization (MR) can further use pQTLs as genetic proxies to elucidate the causal impact of protein level on disease pathogenesis and speed up drug development.

In this study, we screened 996 plasma proteins related to endothelial function and inflammation and examined their associations with SVD using a 2-sample MR approach. We studied symptomatic LS and 5 MRI markers of SVD severity; (1) WMH volume measured from T2-weighted fluid-attenuated inversion recovery images, (2) mean diffusivity (MD; a diffusion tensor imaging metric of the degree of water diffusion in the brain), (3) fractional anisotropy (FA; a diffusion tensor imaging measure of the directionality of diffusion), (4) CMB, and (5) EPVS in white matter. The 2 diffusion tensor imaging measures FA and MD have been shown to be very sensitive to diffuse white matter ultrastructural damage in SVD^10^ and to correlate with cognition in SVD more strongly than WMH.^11^ Among the candidate proteins identified from the MR, we performed colocalization analyses to strengthen the evidence. We further examined the candidate proteins in their associations with cognitive performance, all-cause dementia, and all-cause stroke in the UK Biobank. Lastly, we explored the therapeutic potential of the candidate proteins for SVD treatment.

## Methods

The study is reported following the STROBE (Strengthening the Reporting of Observational Studies in Epidemiology) guidelines for observational and Mendelian randomization studies (Supplemental Material).

### Data Availability

Data from the UK Biobank are available to researchers through application at http://www.ukbiobank.ac.uk/using-the-resource/. The GWAS summary statistics are available on the GWAS Catalogue with accession numbers detailed in the Supplemental Methods.

### Study Populations of the Proteomic Data

The UKB (UK Biobank) recruited over 500 000 participants from 2006 to 2010. Their blood was sampled for genotype and biomarker assessments. From 2020 to 2021, the UKB-PPP (UKB Pharma Proteomics Project) randomly retrieved 46 595 blood samples collected at baseline to undergo proteomic assessment covering 2922 proteins using the Olink Explore 3072 Assay. For the proteome-wide GWAS, 34 557 participants with European ancestry formed the discovery cohort, while the remaining participants became the replication cohort. The detailed procedures of proteomic profiling and data processing have been reported by Sun et al.^8^ The Iceland 36K is a population-based study involving 35 892 Icelanders recruited from 2000 to 2019. The participants’ plasma samples were measured with the SomaScan version 4 Assay, capturing 4670 proteins. The detailed study protocol has been published by Eldjarn et al.^9^

### Mendelian Randomization

A hypothesis-driven approach was used to select candidate proteins to be screened using MR. Literature reviews were performed to identify proteins involved in endothelial dysfunction, inflammation, blood-brain barrier breakdown, oxidative stress, neuro-glia-vascular unit, and vascular remodeling in the context of SVD,^12,13^ vascular cognitive impairment,^14^ dementia-causing diseases,^15,16^ and cardiovascular diseases^17^ (Supplemental Methods; Table S1). Their genetic data availability was checked among the 5758 nonoverlapped assays analyzed in the UKB-PPP and Iceland 36K studies, using a keyword-based search strategy adapted from Lindbohm et al.^15^ In addition to the proteins selected from the literature review, we included all 736 protein assays from the Olink Inflammation Panel I and II. After removing duplicates, we filtered the selected proteins based on the quality and feasibility of their genetic data with the criteria defined a priori (Figure S1). In total, 996 protein assays were prioritized for MR.

GWAS summary statistics for the proteins were extracted from the UKB-PPP discovery cohort or the Iceland 36K study normalized set. For overlapping assays, those from the UKB-PPP were utilized to maintain consistency between the MR analysis and the regression analyses performed within the UKB cohort. The summary statistics for WMH (n=55 291), MD (n=36 460), and FA (n=36 533) were obtained from Koohi et al.^18^ The summary statistics for CMB (n=3556 cases of any brain microbleeds, 22 306 controls) were obtained from Knol et al.^19^ The summary statistics for EPVS in white matter (n=9324 cases, 29 274 controls) were obtained from Duperron et al.^20^ The summary statistics for LS (n=6030 cases, 248 929 controls) diagnosed with TOAST (Trial of ORG 10172 in Acute Stroke Treatment) criteria^21^ or MRI evidence were obtained from Traylor et al.^22^ In addition, because MRI phenotyping of LS is more accurate, we performed a secondary analysis using a GWAS on 3199 exclusively MRI-confirmed LS cases, comprising 2612 cases from Traylor et al^22^ and 587 additional cases.^18^ GWAS summary statistics for individuals of European ancestry were used for all proteins and outcomes except for CMB, which included 3% of participants from other ancestries. Further details of the outcome of GWAS are provided in the Supplemental Methods and Table S2.

Uncorrelated single nucleotide polymorphisms (SNPs; *r*^2^<0.01) in cis association with the proteins (±1 Mb of the gene-coding region)^8,9^ and below genome-wide significance level (*P*<5×10^−8^) were eligible as MR instruments. Nineteen of the total 5976 pairs (996 proteins×6 outcomes) could not be tested because neither the instrument nor its proxy existed in the outcome GWAS (Supplemental Methods). Overall 5957 pairs were tested using the TwoSampleMR (version 0.6.8) and MendelianRandomization (version 0.9.0) *R* packages. The fixed-effect inverse-variance weighted method was used when at least 2 instrument SNPs were available. A Wald ratio was calculated when only 1 instrument SNP was present. F statistics were calculated to quantify the strength of the instruments. MR pleiotropy residual sum and outlier tests were performed to identify possible horizontal pleiotropy. A false discovery rate (FDR) threshold of 5% was used to control for multiple testing across the 6 outcomes.

Four sensitivity analyses were performed among the proteins identified with causal evidence from the primary MR (Supplemental Methods). First, the instruments were changed from those selected based on linkage disequilibrium clumping to independent cis pQTLs derived from the conditional analyses by Sun et al^8^ or Eldjarn et al.^9^ Second, to mitigate the possibility of confounding by linkage disequilibrium, additional MR tests were performed to assess the associations of neighboring proteins of the candidates with SVD. Third, an external replication analysis was conducted using the overlapped assays on the SomaScan platform with the same MR approach. Fourth, multivariable MR was performed to estimate the direct effect of each protein conditional on systolic blood pressure,^23^ a major risk factor for SVD.^24^

### Colocalization

Pairwise colocalization was performed to identify shared genetic variants coregulating the plasma level of each candidate protein and the 6 outcomes using the coloc (version 5.2.3) *R* package. To minimize false positives, each genetic region was narrowed down to ±200 kb window surrounding the protein-coding gene. A sensitivity analysis was performed using the ±1 Mb cis window. The default priors (*p*_1_=1×10^−4^, *p*_2_=1×10^−^^4^, *p*_12_=1×10^−^^5^) were used. A posterior probability (PP) threshold of H_4_>0.8 was defined. For the pairs with high H_3_ PP, conditional colocalization was conducted using the coloc.susie *R* function in case true colocalizing signals were masked by the presence of multiple association signals in the region.

In addition, we examined whether gene expression of the candidate proteins had been identified in brain cells or peripheral blood mononuclear cells (PBMCs) in reference to the findings of Bryois et al^25^ and Yazar et al^26^ (Supplemental Methods). For the proteins with gene expression found in these cells, we further queried if their cis single-cell expression QTL (sc-eQTL) had been identified. If so, we matched their cis sc-eQTLs to the same loci in the GWAS summary statistics of the proteins and SVD traits. We then reviewed and reported the associations between these genetic loci and the plasma protein levels or SVD traits.

### Associations With Cognition, Dementia, and Stroke

Epidemiological analyses were performed in the UKB-PPP cohort to investigate whether circulating protein abundance was associated with baseline cognitive performance or future risk of all-cause dementia or any stroke during the prospective follow-up. Prevalent dementia or stroke cases at baseline were excluded. The baseline values of normalized protein expression were obtained for the 13 candidate proteins identified as causal from the primary MR. They were further inverse-rank normalized to minimize outlier effects and ensure comparability across proteins.^8^ Individuals with missing normalized protein expression values were excluded from the analysis on a protein-by-protein basis.

Two sets of secondary analyses were performed, with covariate characterization and modeling detailed in the Supplemental Methods. First, for both the cross-sectional and survival analyses, we additionally adjusted for Townsend deprivation index (continuous), body mass index (kg/m^2^), smoking (current/not current), alcohol drinking (current/not current), systolic blood pressure (mm Hg), total cholesterol (mmol/L), LDL (low-density lipoprotein) cholesterol (mmol/L), and baseline diabetes (yes/no). Education (years) was also adjusted when the outcomes were cognitive tests. Second, we performed mediation analyses estimating (1) the direct effect of each protein on the risk of dementia or stroke and (2) the proportion of mediation by systolic blood pressure, adjusting for the same set of covariates as in the primary analyses. Overall, we used data from baseline assessment for all covariates where possible. If an individual was missing data from the baseline visit for a particular covariate, we used data from the earliest available repeat assessment for that individual if available. Participants with missing values for any of the covariates and any of the 4 outcomes were excluded (n=4505, ≈8.7% of the UKB-PPP cohort). Multiple testing was corrected with an FDR threshold of 5%, accounting for the 13 candidate proteins identified from the MR analysis with each outcome.

#### Cross-Sectional Analysis on Cognition

At baseline, all participants were invited to complete reaction time and pairs matching tests as measures of processing speed and visuospatial memory, respectively (Supplemental Methods).^27^ These 2 test scores were used as outcomes for the cross-sectional analyses, with higher points indicating longer reaction time or more matching errors. Both cognitive test scores were highly skewed. Therefore, reaction time was natural log-transformed before being fitted in the multivariable linear regression, and the number of pair-matching errors was modeled using negative binomial regression because this value was discrete and zero-inflated. Based on the coefficient estimates, we calculated the % change in reaction time or matching errors per 1-unit increase in each protein’s normalized value. The primary models were adjusted for age and sex.

#### Survival Analysis on Dementia and Stroke

All-cause dementia and all-cause stroke were used as the outcomes (Supplemental Methods), which were ascertained by the UKB algorithm based on linked healthcare records and death certificates.^28,29^ Fine-Gray models were applied to estimate the subdistribution hazard of dementia or stroke per 1-unit increase in each protein’s normalized value. For dementia, death from all other causes, including acute stroke, was accounted for as a competing risk and vice versa for stroke. As a sensitivity analysis, we also estimated cause-specific hazard ratios using Cox-proportional hazards models by treating death from other causes as a censoring mechanism. The proportional hazards assumption was examined by plotting the Schoenfeld residuals for each protein. To control for confounding, we adjusted for age and sex (male/female) in the primary models for stroke, and we controlled for age, sex, education (years), and APOE (apolipoprotein E) ε4 carrier status (yes/no) in the primary models for dementia. Age was modeled as linear and quadratic terms.

### Curation Using DrugBank Databases

GREP (Genome for Repositioning Drugs) software was used to quantify the enrichment of protein candidates among the drug targets of clinical indication classes, including the *International Classification of Diseases, Tenth Revision* (*ICD-10*) and the Anatomic Therapeutic Chemical classification. The drug targets queried in GREP cover the approved or investigated drugs in the DrugBank and the Therapeutic Target databases. We additionally searched each protein candidate in the DrugBank to obtain drug information on the developmental status and the mechanisms of action.

## Results

### Identification of Endothelial and Inflammatory Proteins Associated With SVD

**Figure 1.**
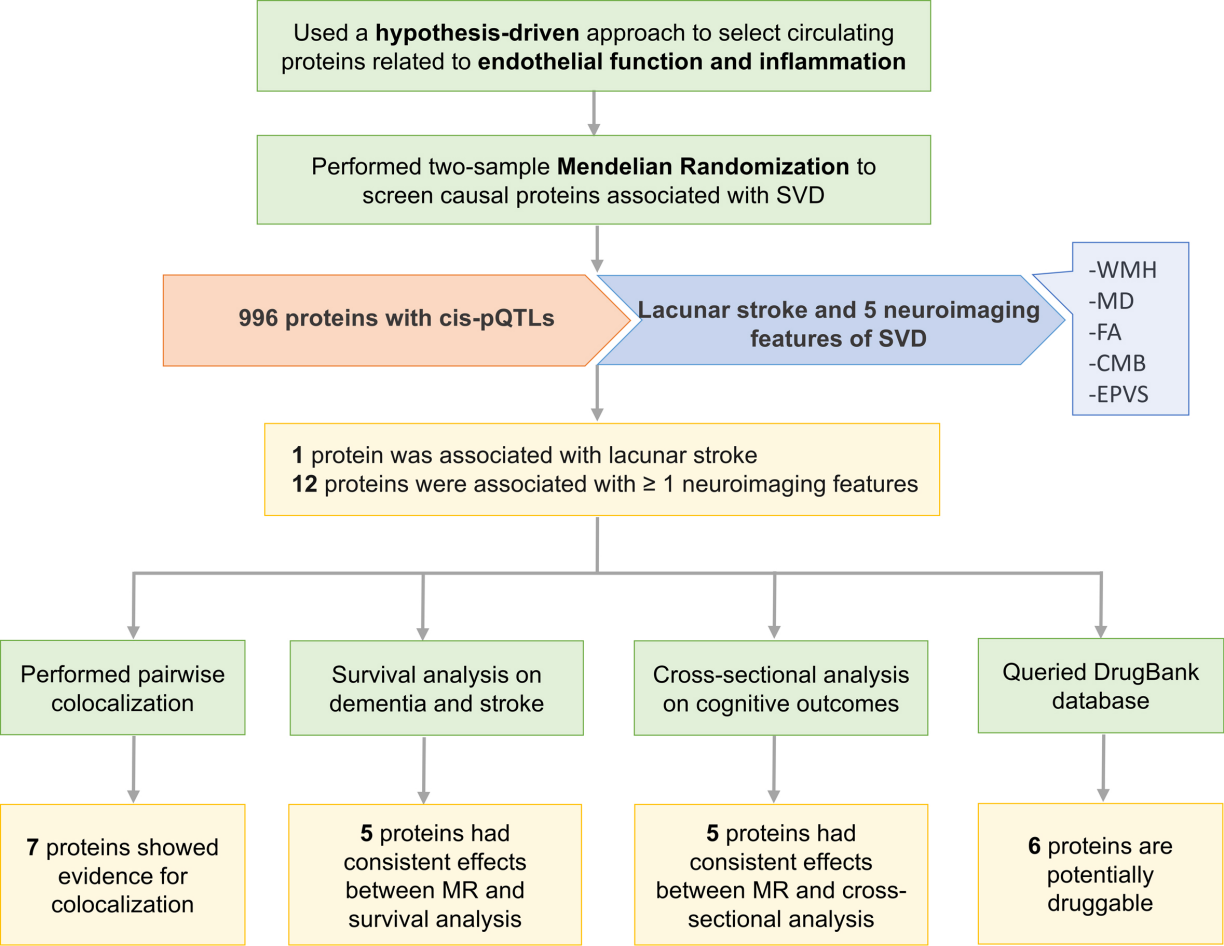
**Study workflow.** CMB indicates cerebral microbleeds; EPVS, enlarged perivascular space; FA, fractional anisotropy; MD, mean diffusivity; MR, Mendelian randomization; pQTL, protein quantitative trait loci; SVD, small vessel disease; and WMH, white matter hyperintensity.

**Figure 2.**
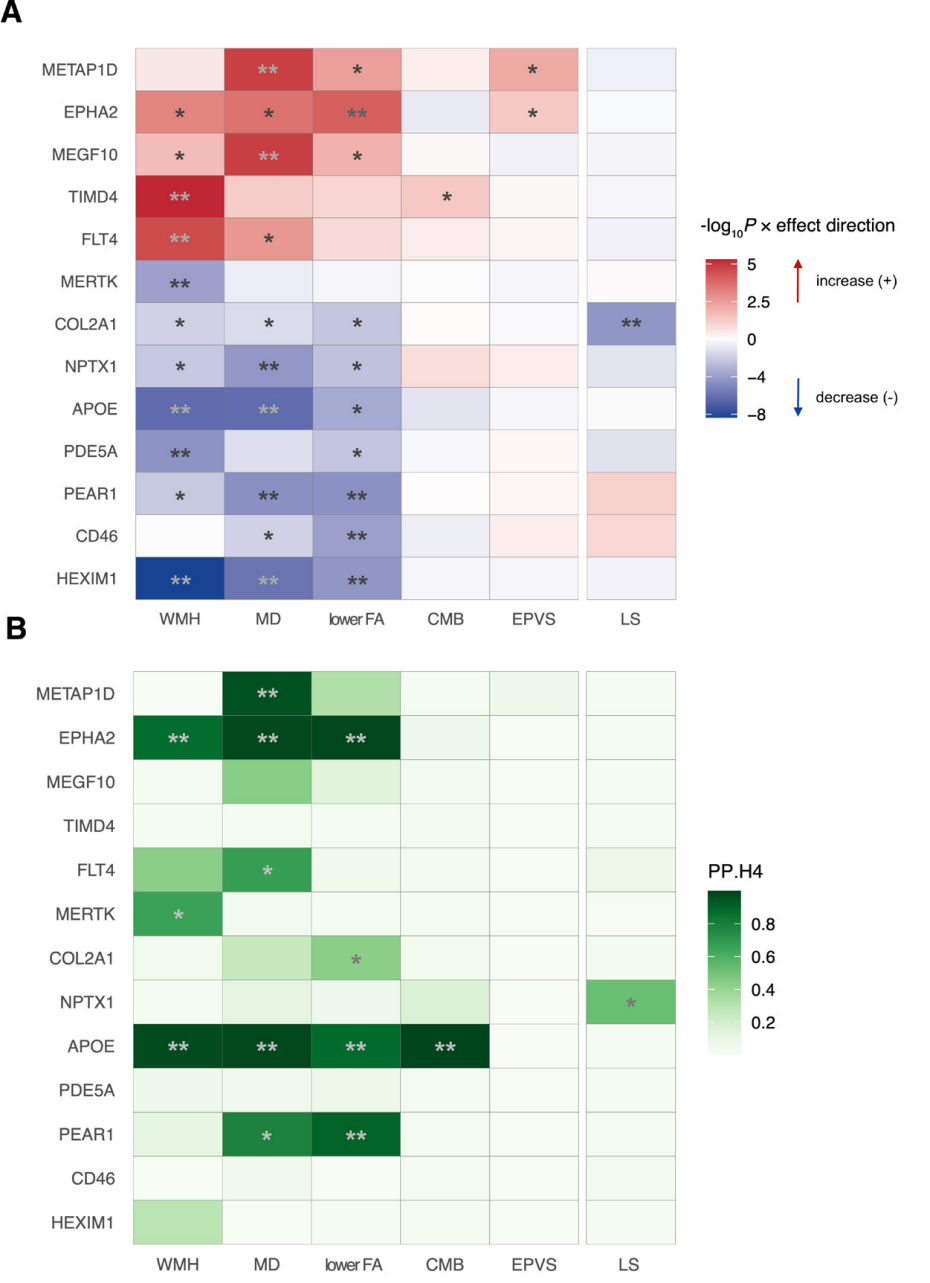
**Heatmaps showing the proteins that were associated with ≥1 small vessel disease (SVD)–related outcomes. A**, Mendelian randomization results from the primary analysis. Red color indicates an increased risk while blue color suggests a decreased risk per 1-unit increase in the normalized expression level of the circulating protein. The color shade corresponds to the strength of the *P* values, with darker color indicating stronger evidence for a causal association. False discovery rate (FDR) was calculated via the Benjamin-Hochberg method to account for multiple testing across the 6 outcomes. A total of 17 protein-outcome pairs, covering 13 unique proteins, demonstrated significant associations below an FDR of 5%. **FDR-corrected *P*<0.05,* unadjusted *P*<0.05. All genetic instruments were identified in *cis* association with the protein below a genome-wide *P* threshold of 5×10^−8^. **B**, Pairwise colocalization results. Color shade indicates the posterior probability (PP) of both traits sharing a single causal variant (ie, PP.H_4_). **PP.H_4_>0.8; *PP.H_4_>PP of any other hypothesis. CD46, membrane cofactor protein; CMB, cerebral microbleeds; COL2A1, collagen type II α-1 chain; EPHA2, ephrin type-A receptor 2; EPVS, enlarged perivascular space; FA, fractional anisotropy; FLT4, vascular endothelial growth factor receptor 3; HEXIM1, hexamethylene bis-acetamide-inducible protein 1; LS, lacunar stroke; MD, mean diffusivity; MEGF10, multiple epidermal growth factor-like domains protein 10; MERTK, tyrosine-protein kinase Mer; METAP1D, methionine aminopeptidase 1D, mitochondrial; NPTX1, neuronal pentraxin-1; PDE5A, cGMP-specific 3',5'-cyclic phosphodiesterase; PEAR1, platelet endothelial aggregation receptor 1; TIMD4, T-cell immunoglobulin and mucin domain–containing protein 4; and WMH, white matter hyperintensity.

For each of the 996 proteins involved in endothelial function and inflammation, MR was used to evaluate its association with LS and 5 neuroimaging markers (Figure 1). The primary analysis was performed for 5957 protein-outcome pairs (Supplemental Material 1). Seventeen pairs (0.285%) were significant after multiple testing correction at 5% FDR, corresponding to a *P* threshold of 1.4×10^−^^4^, 2-sided. All these pairs had strong instruments (F statistics > 10; Supplemental Material 2). MR pleiotropy residual sum and outlier test did not identify substantial horizontal pleiotropy for any of these pairs (Supplemental Material 3). Of the 17 pairs covering 13 unique proteins, 9 proteins were associated with 1 imaging feature of SVD; 3 proteins (APOE, PEAR1 [platelet endothelial aggregation receptor 1], and HEXIM1 [hexamethylene bis-acetamide-inducible protein 1]) were associated with ≥2 neuroimaging markers; 1 protein (COL2A1 [collagen type II α-1 chain]) was associated with LS (Figure 2A). Coherent results were detected across features of high WMH volume, high MD, and low FA, all 3 suggesting white matter pathology. When the LS cases were restricted to those confirmed by MRI, no significant result was found after multiple testing correction at 5% FDR (Supplemental Material 4). The association between COL2A1 and MRI-confirmed LS was not significant, although its effect was consistent with that in the primary result (odds ratio_MRI-confirmed LS_, 0.94 [95% CI, 0.86–1.03]; *P*=0.18 versus odds ratio_LS primary_, 0.89 [95% CI, 0.86–0.91]; *P*=5×10^−5^).

Sensitivity analyses were conducted among the 13 candidate proteins identified from the MR. First, the instruments were changed to the conditionally independent cis pQTLs reported by Sun et al.^8^ All pairs remained significant (*P*<0.05, 2-sided) except for the CD46 (membrane cofactor protein)—lower FA pair (Figure S2). Second, the protein-coding genes situated within ±200 kb of the 13 candidate genes were identified with the LocusZoom plots (Supplemental Methods). Sensitivity analysis indicated that HAVCR2 (hepatitis A virus cellular receptor 2) was associated with WMH and EPVS, and CR1 (complement receptor type 1) was associated with MD and lower FA, resembling those identified for TIMD4 (T-cell immunoglobulin and mucin domain–containing protein 4) and CD46 in the primary results, respectively (Figure S3). Third, external replication was performed for 10 of the 13 candidate proteins overlapped between the Olink and SomaScan platforms (Supplemental Material 5). Nine of these showed consistent results while the association for 1 protein, NPTX1 (neuronal pentraxin-1), attenuated although its effect direction remained consistent (Figure S4). Fourth, in the multivariable MR, 10 of the 13 proteins showed significant and consistent effects as in the primary MR (Supplemental Material 6). The associations for 2 proteins (PDE5A [cGMP-specific 3',5'-cyclic phosphodiesterase] and CD46) were no longer significant, but their effect directions remained consistent (Figure S5). The association of HEXIM1 with white matter also attenuated; however, it showed a positive association with CMB.

### Shared Genetic Associations Between Candidate Proteins and SVD

Among the 13 candidate proteins identified from the MR results, 4 proteins (METAP1D [methionine aminopeptidase 1D, mitochondrial], EPHA2 [ephrin type-A receptor 2], APOE, and PEAR1) were identified with genetic variants that coregulate their plasma abundance and SVD traits (colocalization PP.H_4_>0.8; Figure 2B; Supplemental Material 7). Of the 17 pairs found significant by the MR, 6 pairs (covering the 4 proteins) were colocalized and 1 pair MERTK (tyrosine-protein kinase Mer)-WMH showed a moderate probability of hypothesis 4 (PP.H_4_=0.65). Five pairs (involving TIMD4, PDE5A, FLT4 [vascular endothelial growth factor receptor 3], NPTX1, and COL2A1) were assigned with a high PP for hypothesis 1, possibly due to a lack of power (Figure S6). Five other pairs (HEXIM1-WMH, HEXIM1-MD, HEXIM1–lower FA, MEGF10 [multiple epidermal growth factor-like domains protein 10]-MD, and CD46-lower FA) showed a high PP for hypothesis 3. For these 5 pairs, conditional colocalization was conducted in the Sum of Single Effects regression framework in case true colocalizing signals were masked by the presence of multiple association signals in the region. All 5 pairs were identified with conditional signals (Supplemental Material 8). The sensitivity analysis using ±1 Mb window showed consistent results (Supplemental Material 9). Taken together, 7 of the 13 proteins were colocalized with ≥1 SVD traits (Figure S6).

Referencing the single-cell sequencing studies,^25,26^ we found that gene expression was detected for 9 proteins in brain cell types and 7 proteins in PBMCs (Tables S3 and S4). Among them, cis sc-eQTLs had been identified for PDE5A and CD46 in excitatory neurons, inhibitory neurons, and oligodendrocytes. CD46 had sc-eQTLs found in oligodendrocyte precursor cells. Four proteins (TIMD4, FLT4, HEXIM1, and METAP1D) had cis sc-eQTLs identified in CD4 naive/central memory T cells. The sc-eQTLs for TIMD4, FLT4, HEXIM1, CD46, and PDE5A were strongly associated with their plasma levels; *P* values for the SNP-protein associations ranged from 1×10^−^^5^ to 1×10^−^^58^ based on the protein GWAS conducted in the UKB-PPP study (Table S5). However, only one sc-eQTL (rs4632173 for the expression of HEXIM1 in CD4 naive/central memory T cell) showed a strong association with SVD traits (SNP-WMH association: *P*=1×10^−^^8^; SNP-MD association: *P*=1×10^−^^4^; Table S5).

### Circulating Protein Levels in Association With Cognition, Dementia, and Stroke

Baseline characteristics of the study population are presented in Table S6. Briefly, among the 47 571 participants included in our analysis, 1228 (2.6%) had developed all-cause dementia and 1268 had experienced any stroke (2.7%) at the time of our data extraction in November 2023. The average time to dementia and stroke was 9.2 and 8.2 years, respectively. The average follow-up was 14.0 years among the overall participants.

**Figure 3.**
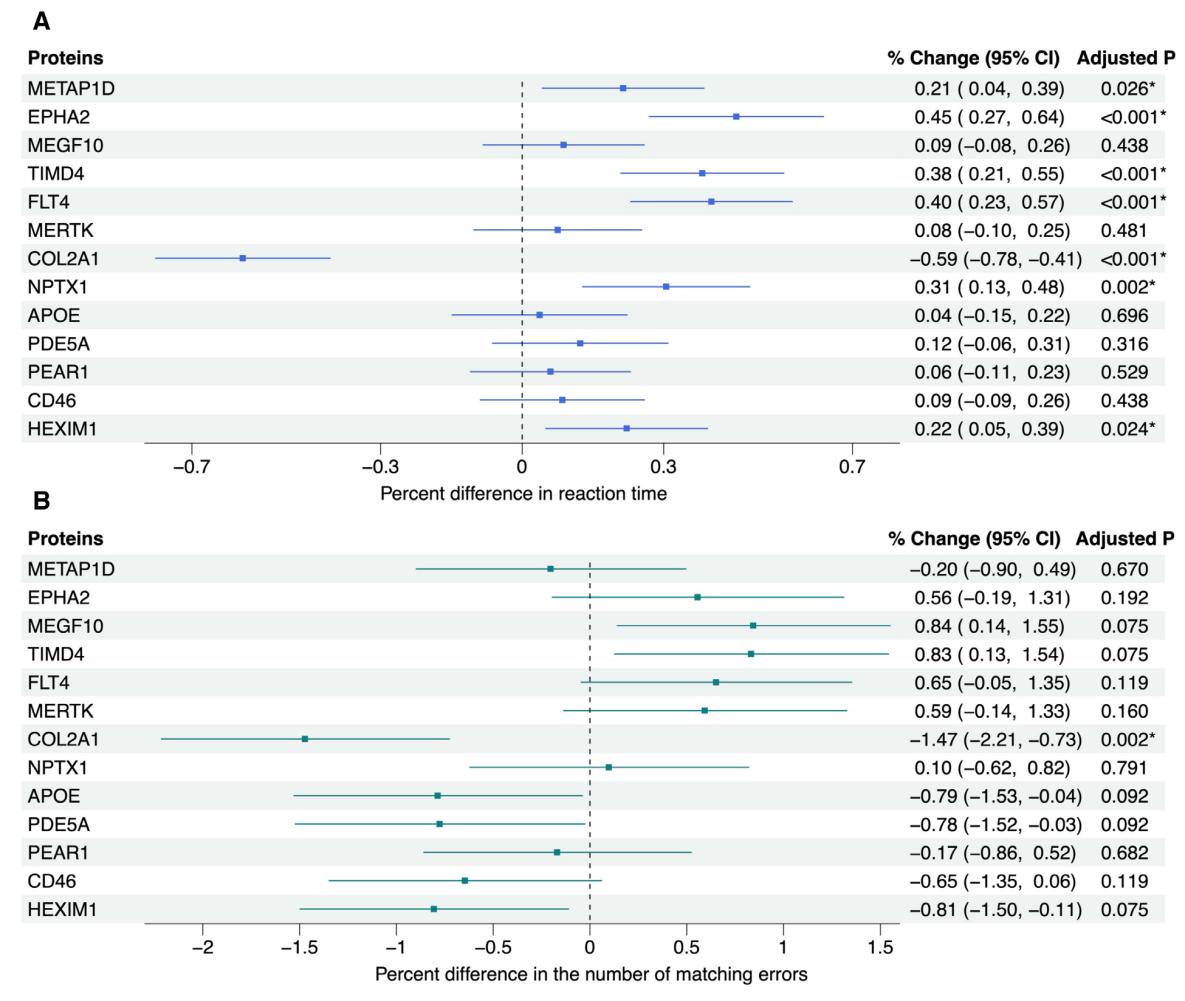
**Plots showing estimates for the association between each candidate protein and cognitive functions as measured by reaction time and pairs matching tests at the UK Biobank baseline.** Multivariable linear regression and negative binomial regression were used to estimate the percent difference in reaction time and matching errors per 1-unit increase in the inverse-normal transformed normalized protein expression value of each protein, respectively. For both cognitive outcomes, the models were adjusted for sex and age at baseline. **A**, The % change in reaction time. **B**, The % change in matching errors. *False discovery rate–corrected *P*<0.05. CD46 indicates membrane cofactor protein; COL2A1, collagen type II α-1 chain; EPHA2, ephrin type-A receptor 2; FLT4, vascular endothelial growth factor receptor 3; HEXIM1, hexamethylene bis-acetamide-inducible protein 1; MEGF10, multiple epidermal growth factor-like domains protein 10; MERTK, tyrosine-protein kinase Mer; METAP1D, methionine aminopeptidase 1D, mitochondrial; NPTX1, neuronal pentraxin-1; PDE5A, cGMP-specific 3',5'-cyclic phosphodiesterase; PEAR1, platelet endothelial aggregation receptor 1; and TIMD4, T-cell immunoglobulin and mucin domain–containing protein 4.

In cross-sectional analyses adjusted for age and sex, increasing plasma abundance of 6 proteins (METAP1D, EPHA2, TIMD4, FLT4, NPTX1, and HEXIM1) were associated with prolonged reaction time (FDR-corrected *P*<0.05; Figure 3A). Conversely, a 1-unit increase in COL2A1 abundance was associated with shorter reaction time (% change, −0.59% [95% CI, −0.78% to −0.41%]) and fewer matching errors (% change, −1.47% [95% CI, −2.21% to −0.73%]; Figure 3A and 3B). No other significant associations were observed for the pairs matching test (Figure 3B). Of the 7 proteins that showed a significant effect on either cognitive test, 5 had consistent effects with those identified in the MR analysis (COL2A1, METAP1D, EPHA2, TIMD4, and FLT4). Adjusting for demographic and vascular risk factors in the secondary models did not meaningfully change the results from the primary models (Supplemental Material 10).

**Figure 4.**
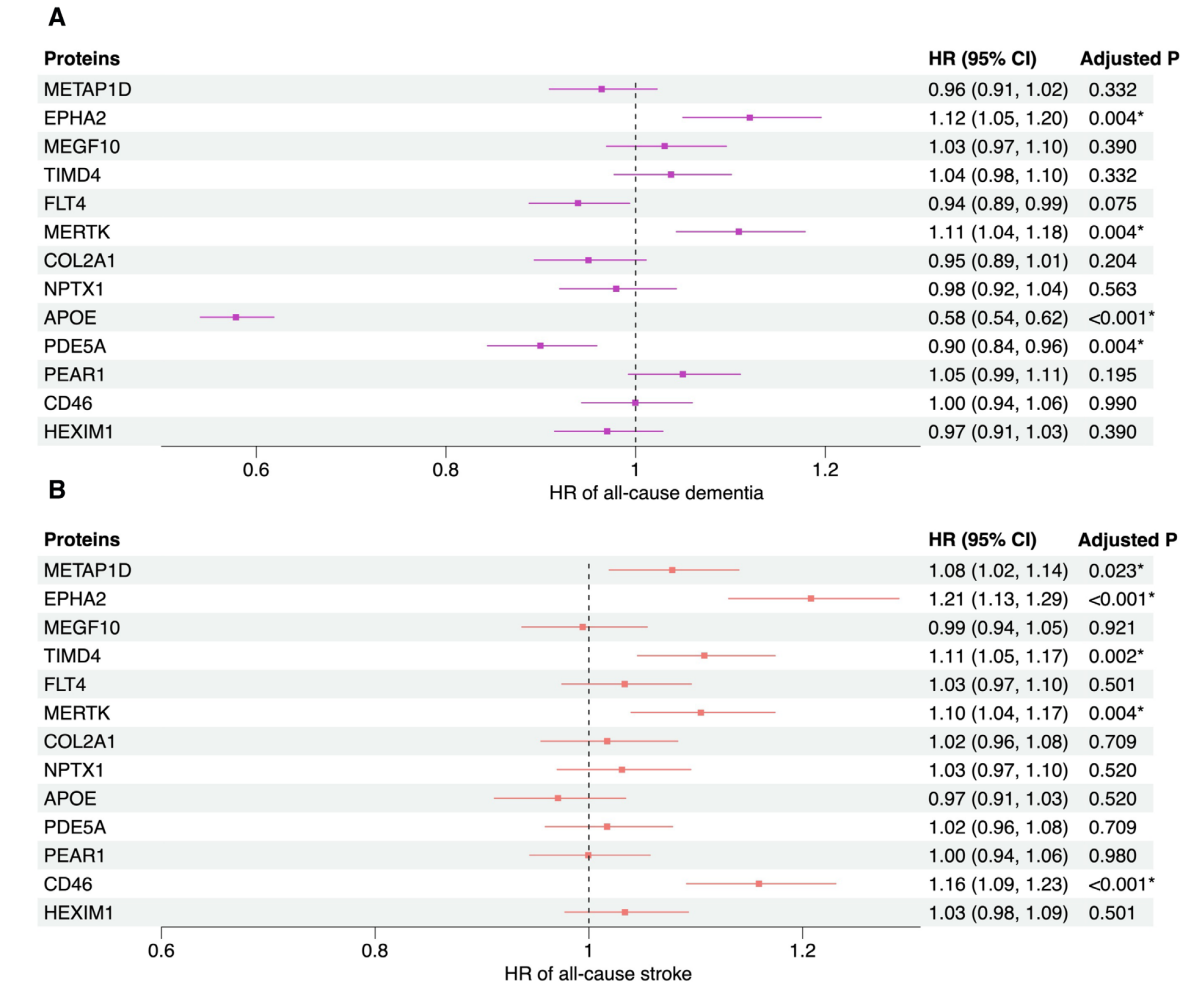
**Plots showing estimates for the association between candidate proteins and all-cause dementia and all-cause stroke.** Fine-Gray model estimated the hazard ratios (HR) of incident dementia or stroke per 1-unit increase in the inverse-normal transformed normalized protein expression value of each protein after competing risk was accounted for. For dementia, the models were adjusted for age, sex, education in years, and APOE ε4 carrier status. For stroke, the model was adjusted for age and sex. **A**, All-cause dementia as the outcome. **B**, All-cause stroke as the outcome. *False discovery rate–corrected *P*<0.05. CD46 indicates membrane cofactor protein; COL2A1, collagen type II α-1 chain; EPHA2, ephrin type-A receptor 2; FLT4, vascular endothelial growth factor receptor 3; HEXIM1, hexamethylene bis-acetamide-inducible protein 1; MEGF10, multiple epidermal growth factor-like domains protein 10; MERTK, tyrosine-protein kinase Mer; METAP1D, methionine aminopeptidase 1D, mitochondrial; MR, Mendelian randomization; NPTX1, neuronal pentraxin-1; PDE5A, cGMP-specific 3',5'-cyclic phosphodiesterase; PEAR1, platelet endothelial aggregation receptor 1; and TIMD4, T-cell immunoglobulin and mucin domain–containing protein 4.

In the survival analyses with the Fine-Gray models using an FDR-corrected *P*<0.05 as the threshold, 4 of the 13 protein candidates were associated with all-cause dementia (EPHA2, APOE, PDE5A, and MERTK) after adjusting for age, sex, education, and APOE ε4 carrier status and 5 (METAP1D, EPHA2, TIMD4, MERTK, and CD46) were associated with any stroke conditioning on age and sex (Figure 4A and 4B). EPHA2 was significantly associated with both dementia and stroke, and their effect directions were consistent with the MR findings. Although significant results were also identified for MERTK with both outcomes and for CD46 with stroke, their hazard ratios suggested opposite effects to those found in the MR analysis. The other 4 proteins (APOE, PDE5A, METAP1D, and TIMD4) were associated with either dementia or stroke with consistent effects as the MR analysis. Consistent results were observed across the primary Fine-Gray models, the secondary models adjusting for demographic and vascular risk factors (Supplemental Material 11) and the cause-specific Cox models (Supplemental Material 12). Visual examination of the Schoenfeld residuals for each protein did not identify significant violation of the proportional hazards assumption.

In the mediation analyses, the significant protein-outcome pairs identified from the survival analyses all remained significant (FDR<0.05) in terms of their direct effects except METAP1D (Supplemental Material 13). For any protein-dementia pair, the proportion of mediation by systolic blood pressure was not significant; for all protein-stroke pairs, the proportions of mediation were only moderate with a range of −23% to 30%, despite their significance (Supplemental Material 13).

### Druggability of Candidate Proteins

Of the 13 proteins, 6 (COL2A1, EPHA2, FLT4, MERTK, PDE5A, and APOE) have been investigated and tested as drug targets (Table). We conducted an analysis using GREP software, which indicated enrichments in antineoplastic and immunomodulating agents targeting EPHA2, FLT4, and MERTK (Supplemental Material 14 and 15). Moreover, PDE5A was enriched as a therapeutic target for diseases affecting cardiovascular, respiratory, and genitourinary systems. In the DrugBank database, collagenase clostridium histolyticum, a drug that was under investigation, was found to regulate COL2A1 levels. Supplements that affect zinc availability were also shown to modulate APOE level via their interactions; however, their effects may not be specific. The medications targeting these proteins, their mechanisms of action, and their developmental status are summarized in the Table.

**Table.**
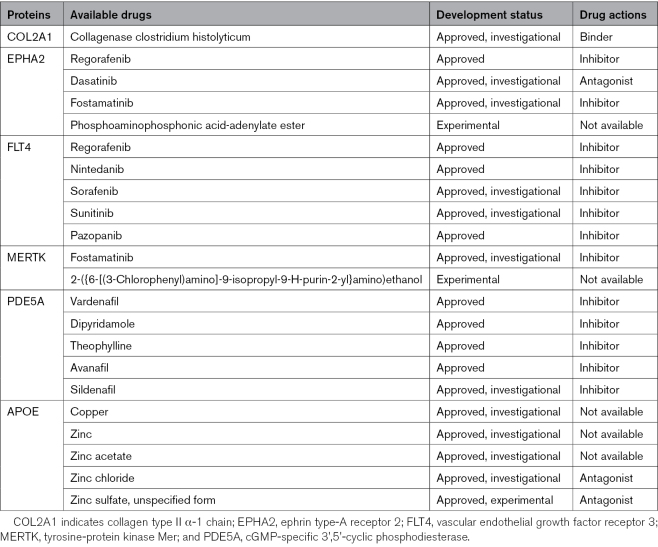
Candidate Proteins Targeted by Existing Drugs

**Figure 5.**
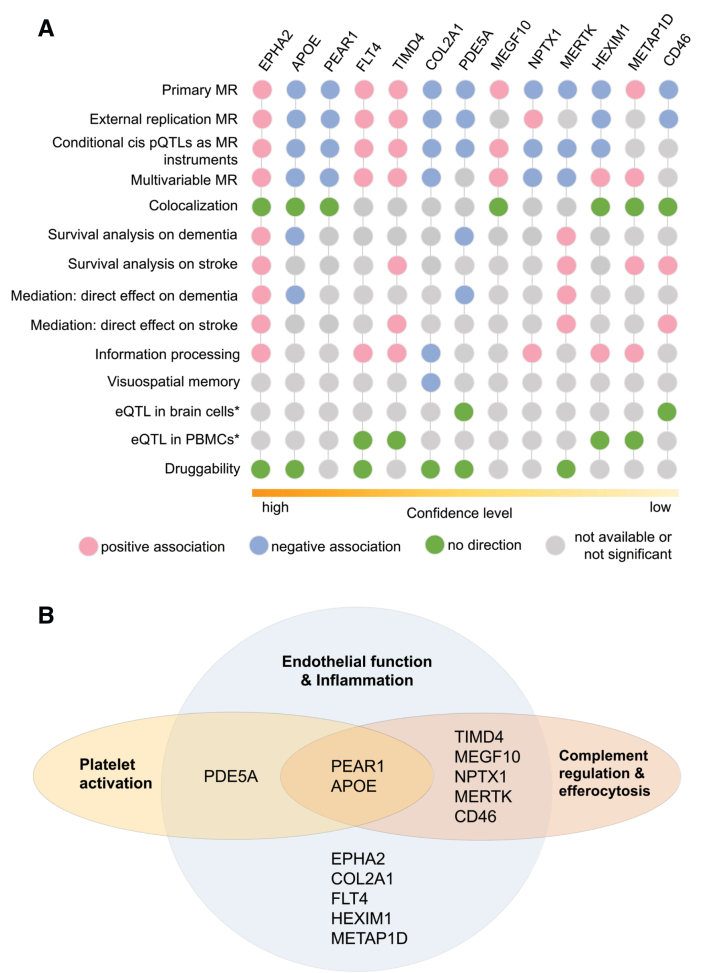
**A summary of evidence among the 13 protein candidates. A**, Analysis results across multiple methods. *The results were referenced from prior studies. **B**, Functional categorization of the 13 protein candidates. CD46, membrane cofactor protein; COL2A1, collagen type II α-1 chain; EPHA2, ephrin type-A receptor 2; eQTL, expression quantitative trait loci; FLT4, vascular endothelial growth factor receptor 3; HEXIM1, hexamethylene bis-acetamide-inducible protein 1; MEGF10, multiple epidermal growth factor-like domains protein 10; MERTK, tyrosine-protein kinase Mer; METAP1D, methionine aminopeptidase 1D, mitochondrial; NPTX1, neuronal pentraxin-1; PBMC, peripheral blood mononuclear cell; PDE5A, cGMP-specific 3',5'-cyclic phosphodiesterase; PEAR1, platelet endothelial aggregation receptor 1; pQTL, protein quantitative trait loci; TIMD4, T-cell immunoglobulin and mucin domain–containing protein 4.

The results for the 13 proteins are summarized in Figure 5A in the order of descending level of confidence. A Venn diagram illustrates the 13 proteins according to their roles in different pathways underlying endothelial function and inflammation (Figure 5B).

## Discussion

Within a hypothesis-driven framework, a total of 996 proteins related to endothelial dysfunction and inflammation were assessed in their associations with SVD using MR. MR evidence supported 1 protein (COL2A1) associated with LS and 12 additional proteins (EPHA2, APOE, PEAR1, FLT4, TIMD4, PDE5A, MEGF10, MERTK, NPTX1, HEXIM1, METAP1D, and CD46) associated with ≥1 neuroimaging features of SVD. Colocalization analysis suggested that 7 of the 13 proteins (EPHA2, APOE, PEAR1, MEGF10, HEXIM1, METAP1D, and CD46) shared causal genetic variants with SVD. Using cross-sectional and survival analyses in the UKB-PPP cohort, 7 proteins (COL2A1, EPHA2, APOE, FLT4, PDE5A, TIMD4, and METAP1D) were found to be associated with information processing speed, visuospatial memory, incident all-cause dementia, or incident any stroke, with their effect directions consistent with the MR findings.

We found the most consistent evidence for EPHA2 and APOE with the support of both MR and conventional epidemiological analyses (Figure 5A). Eph receptor and ephrin signaling is involved in proinflammatory gene expression.^30^ In a mouse model of focal stroke, EPHA2 deletion was shown to reduce MMP-9 (matrix metalloproteinase-9) expression and leukocyte infiltration while increasing expression of a tight junction protein, zona occludens-1.^31^ In an endothelial cell line of human brain microvasculature, phosphorylation of EPHA2 receptor was identified to disrupt tight junction.^32^ Both studies were consistent with our findings in which increasing plasma EPHA2 abundance was associated with white matter damage, prolonged reaction time, and increased risks of dementia and stroke. APOE has been studied for decades. Consistent with the prior study,^33^ we found reduced plasma APOE levels were associated with white matter damage, CMBs, and increased risk of all-cause dementia independent of *APOE* genotypes (Figures 2A and 4A). Recent research has found that APOE can inhibit classical complement cascade by binding to C1q (complement component 1q),^34^ pointing to another probable inflammation-mediated pathway to SVD.

We considered PEAR1 as another causal candidate. It had consistent evidence across MR sensitivity and colocalization analyses, although its associations with cognition, dementia, and stroke were not statistically significant. PEAR1, also known as JEDI (Jagged and Delta protein) or MEGF12 (multiple epidermal growth factor-like domains protein 12), mediates the phagocytosis of apoptotic neurons.^35^ Moreover, PEAR1 has been identified as a high-affinity receptor for SVEP1 (Sushi, von Willebrand Factor type-A, EGF, and pentraxin–domain containing 1) protein.^36^ Prior human studies have observed SEVP1 in its associations with inflammation in atherosclerotic plaques, WMH, and dementia.^37^ It will be of interest to investigate whether the interaction between PEAR1 and SVEP1 plays a role in SVD.

We identified FLT4, TIMD4, and COL2A1 as likely candidates. Consistent with the MR analyses, these proteins also showed significant associations with either cognitive performance, dementia, or stroke (Figure 5A). Specifically, FLT4 (ie, VEGFR3) is the receptor for vascular endothelial growth factors C and D. In human carotid artery specimen, FLT4 was found to express in monocytes or macrophages in atherosclerotic lesions, where it could regulate immune cell apoptosis and plaque stability.^38^ Another protein, TIMD4 (ie, Tim-4), functions to mediate efferocytosis and cytokine production together with its genetic neighbors, HAVCR1 (hepatitis A virus cellular receptor 1; ie, Tim-1) and HAVCR2 (ie, Tim-3).^39^ Our MR sensitivity analysis also showed that both TIMD4 and HAVCR2 might be associated with WMH. Lastly, collagen type II α-1 chain (COL2A1) is an extracellular matrix protein whose protein family members, COL4A1 and COL4A2 (collagen type-IV α-1 and α-2 chains), have been shown to play essential roles in SVD pathogenesis.^40^

We found less consistent evidence for the proteins PDE5A, MEGF10, MERTK, and NPTX1. However, each of them was supported by at least 3 of the methods and involved in relevant biological processes. PDE (phosphodiesterase) has been shown to regulate the activation of platelets and their interaction with inflamed endothelial cells.^41^ MEGF10 mediates efferocytosis as a receptor for C1q which signals apoptosis.^42^ Interestingly, another candidate, MERTK can collaborate with both TIMD4 and MEGF10 on efferocytosis.^43,44^ NPTX1 together with its family members NPTX2 and NPTX receptor has also been implicated in complement regulation^45^ and cognitive impairment.^46^

Despite being identified from the primary MR, HEXIM1, METAP1D, and CD46 were subject to further validation. The gene *HEXIM1* is located downstream of *PLCD3 (Phospholipase C-delta-3*), which has been mapped to a GWAS signal for blood pressure.^47^ Further research could examine whether it is the HEXIM1-regulated inflammation or PLCD3-linked hypertension that is causal for SVD or whether they correspond to different SVD mechanisms. METAP1D is upstream of metabolism of homocysteine, a marker of vascular inflammation.^5^ Although METAP1D showed consistent results across the primary MR, colocalization, and epidemiological analyses, it only had 1 instrument SNP so it could not be examined in some MR sensitivity analyses. CD46 showed opposite effects between the MR and the regression analyses, whereas its sensitivity analyses indicated null results. However, its genetic neighbor, CR1, showed associations with MD and FA, although they did not pass the FDR threshold in the primary MR. Intriguingly, CD46 and CR1 belong to the same complement pathway,^48^ and CR1 has been associated with Alzheimer disease risk.^49^ However, whether this pathway is causal for SVD needs further validation.

Of the 13 candidate proteins, 7 (APOE, PEAR1, TIMD4, MEGF10, NPTX1, MERTK, and CD46) are potentially involved in complement regulation and efferocytosis, as well as the downstream regulation of inflammation (Figure 5B). This body of evidence suggests that inhibition of excessive complement activation may be an important pathway to target. Two proteins PDE5A and PEAR1 are involved in the regulation of platelet activation, suggesting that antiplatelet therapies may be beneficial. Six of the 13 proteins have been targeted in pharmaceutical products (Table). Drugs that inhibit COL2A1, MERTK, and PDE5 have been developed; however, the optimal targeted levels of these proteins need to be ascertained. The effects of these proteins may also depend on whether it is lifelong exposure (as proxied by MR) or there are critical windows. EPHA2 and FLT4 have been investigated as promising targets for cancer treatment. Small molecule TKIs (tyrosine kinase inhibitors) for EPHA2 and FLT4, such as dasatinib and regorafenib, have been applied in the clinical setting with good efficacy. However, this class of drugs has been reported with adverse events.^50^ For all the candidate proteins, we must go beyond their plasma level and understand their tissue-specific mechanisms. A targeted modulation instead of a simplified inhibition may help prevent their unintended effects and tailor them to manage SVD.

Our study has several limitations. First, instead of a proteome-wide screen, we used a hypothesis-driven approach to filter the proteins related to endothelial function and inflammation. Although this strategy may introduce bias in favor of the well-studied proteins, our selection methods and criteria were formulated a priori to ensure objectiveness. The number of proteins included in our screen also substantially increased compared with previous studies in SVD. Second, to ensure the proteogenomic data were well quality-controlled and replicable, we filtered the proteins based on their coefficient of variation, percentage below the lower limit of detection, and replicated cis pQTLs using the thresholds defined a priori. Although these exclusion criteria helped to ensure robust results, we may have missed some interesting candidates, particularly the proteins with trans pQTLs or rare variants. Third, due to lack of ancestry-specific data from the prior GWAS, our genetic analyses were performed using samples of European ancestry. The generalizability of our MR findings needs to be examined in other racial and ethnic groups. Fourth, due to lack of power and full access to the summary statistics in the single-cell studies, we were unable to perform formal colocalization tests among the eQTLs, pQTLs, and GWAS signals across brain or immune cell types. However, based on a direct mapping of the sc-eQTLs to the SNPs assessed in the protein and outcome GWAS, we were able to observe concordant signals coregulating a gene’s cell type–specific expression and its plasma protein abundance. This finding further suggests that circulating protein levels were correlated with those in the disease-related cell types.

Our findings suggest the roles of endothelial-platelet function and complement-mediated regulation of inflammation in SVD. Future research is necessary to elucidate the pathogenic pathways influenced by these proteins and evaluate the therapeutic potential of each candidate for SVD treatment.

## Article Information

### Acknowledgments

This study made use of the UK Biobank resource under application number 36509.

### Sources of Funding

This research was supported by a joint grant from the British Heart Foundation (ref: SP/F/22/150028) and Dutch Heart Foundation (project 02-001-2021-B021) to Drs Markus, Mallat, de Leeuw, and Riksen. Infrastructural support was provided by the Cambridge British Heart Foundation Center of Research Excellence (RE/18/1/34212) and the Cambridge University Hospitals National Institute for Health and Care Research (NIHR) Biomedical Research Center (NIHR203312). Dr Harshfield was supported by the Alzheimer’s Society (AS-RF-21-017). Dr Riksen was supported by a CardioVasculair Onderzoek Nederland (CVON) grant from the Dutch Cardiovascular Alliance (DCVA) and Dutch Heart Foundation (CVON2018-27; IN-CONTROL II [Inflammatory Reprogramming by Ageing and Microbiome – Targets for Treatment of Cardiovascular Disease]).

### Disclosures

None.

### Supplemental Material

Supplemental Methods

Tables S1–S6

Figures S1–S6

Supplemental Material Sheets 1–15
