# Supplementary material for: Proteins involved in endothelial function and inflammation are implicated in cerebral small vessel disease
Source: Stroke. Author manuscript; Available in PMC 2025 Mar 1. (PMC7617319; doi:10.1161/STROKEAHA.124.049079)
Supplement: Supplementary file 1 [file EMS202074-supplement-Supplemental_Publication_Material.pdf]

Supplemental Materials

Proteins involved in endothelial function and inflammation are implicated in cerebral small vessel disease

Table of Contents

Supplemental Method **2**

Two-sample Mendelian Randomization (MR) *2*

Cross-sectional and survival analyses *7*

Supplemental Figures **11**

Figure S1. A flowchart showing the selection of circulating proteins. *11*

Figure S2. A plot comparing the MR estimates between two methods for instrument selection *12*

Figure S3. A plot showing the MR estimates of three candidate proteins and their neighbouring proteins *13*

Figure S4. Replication results using summary statistics from the Iceland 36K study *14*

Figure S5. Multivariable MR estimating the effect of individual protein on SVD conditional on SBP *15*

Figure S6. A heatmap of the posterior probability from pairwise colocalization *16*

Supplemental Tables **17**

Table S1. Search terms by pathophysiological processes involved in SVD *17*

Table S2. Sources of GWAS summary statistics for SVD-related outcomes *19*

Table S3. The candidate proteins with their gene expression found in 8 brain cell types *20*

Table S4. The candidate proteins with their gene expression identified in 14 PBMCs *20*

Table S5. The SNP-protein and SNP-outcome associations for the reported sc-eQTLs *21*

Table S6. Baseline characteristics of the UKB-PPP cohort by incident dementia or stroke status *23*

Appendix. The list of 996 proteins *24*

Supplemental Method

Two-sample Mendelian Randomization (MR)

Selection of proteins related to endothelial function and inflammation

Literature reviews were performed to identify relevant proteins. We extracted the full names of the proteins and their coding genes from the literature, constructed search terms based on their key characters and abbreviations, and searched their availability among a total of 5,758 non-overlapped assays available in the UKB-PPP and Iceland 36K studies (Figure S1). All proteins targeted by the Olink Inflammation Panel I and II were additionally included if they did not appear in the search terms. For the overlapped proteins targeted by both the UKB-PPP and the Iceland 36K studies, the results from the UKB-PPP study were applied in the primary analysis to keep consistency between the genetic data used in the MR screen and the protein measures used in the later analyses. After the removal of duplicates, 1,698 Olink assays and 1,114 SomaScan assays were kept.

Next, these relevant proteins were filtered based on their usability. Specifically, to increase the likelihood that each protein would have genetic instruments with minimal horizontal pleiotropy, we excluded the proteins without a *cis* pQTL. Then, to ensure the transportability of our study, we excluded the proteins with cis pQTLs not replicated in either internal or external replication cohorts. Proteins with *cis* pQTLs in the HLA region were also excluded because this region is highly pleiotropic and violates the assumption of MR. We also excluded proteins with low measurement accuracy (i.e. coefficient of variation > 20% and below lower limit of detection (LOD) > 5%[1]) to improve our study validity. Next, we excluded proteins with *cis* pQTLs on sex chromosomes since the outcome GWAS were done for autosomes only. Lastly, we filtered out the proteins without MR instruments satisfying the predefined criteria when its summary statistics were harmonized with any of the outcome datasets. The number of proteins excluded at each step was detailed in Figure S1. Overall,

---

[1] Because the limit of detection was not reported on the SomaScan v4 platform, we were unable to filter SomaScan assays using the criterion of 5% below lower limit of detection (LOD) as we did for the Olink assays. However, compared to antibody-based assays such as the Olink, SomaScan assays have been reported to exhibit higher sensitivity with sample concentrations significantly higher than the background. Prior studies only identified 7 human proteins which had below LOD issues using SomaScan v4.1 platform, and none of these 7 proteins ended up in our screening list.

bridging two platforms and filtering based on data usability, we included a total of 996 unique protein assays for subsequent MR analyses.

GWAS of proteins involved in endothelial dysfunction and inflammation

For the circulating proteins measured with the Olink platform, we extracted their summary statistics from the corresponding GWAS conducted within the UKB-PPP discovery cohort, which comprised of 34,557 participants of European ancestry. For the proteins measured with the SomaScan platform, we obtained their GWAS summary statistics calculated using the SMP-normalized protein levels within the full Iceland 36K cohort.

GWAS of neuroimaging features of SVD

We used neuroimaging features of SVD, including high white matter hyperintensity volume (WMH), high mean diffusivity (MD), low fractional anisotropy (FA), presence of cerebral microbleeds (CMB), and extensive white matter perivascular space burden (EPVS) as outcome measures. The GWAS for WMH (N =55,291) was updated based on a meta-analysis pooling data from the UKB imaging cohort and the CHARGE consortium. The GWAS for the two DTI metrics, MD (N =36,460) and FA (N =36,533), were performed within the UKB imaging cohort. The UKB phenotype data on WMH, FA, and MD were obtained following its release in April 2023 with additional quality control steps. The detailed GWAS methods and their summary statistics were made available by Koohi et al. The GWAS summary statistics for CMB (N cases of any brain microbleeds = 3,556; N controls =22,306) were extracted from the Knol et al. study, which conducted a meta-analysis based on 14 cohorts including the UKB imaging cohort (N cases from UKB = 570). The GWAS summary statistics for EPVS in white matter (N cases = 9,324, N controls = 29,274) were obtained from Duperron et al. Briefly, they performed a meta-analysis for the stroke-free participants recruited from 18 cohorts including the UKB, the CHARGE consortium, and the BRIDGET initiative. They dichotomized the scale of perivascular space burden in a cohort-specific manner, with the cases defined as those in the top quartile of a continuous PVS scale. Of note, the UKB imaging cohort also contributed to this meta-analysis (~ 7,123 out of the 9,324 total cases were from the UKB).

For all neuroimaging outcomes except CMB, we used the GWAS summary statistics for participants with European ancestry. The summary statistics for CMB contained a small sample of people from African and Asian ancestries (637 out of 3,556 total CMB cases) as

the authors did not perform an ancestry-specific analysis. Although the GWAS on protein abundance and neuroimaging outcomes both included some of the UKB participants, we considered our MR analysis as a two-sample design. At the current stage, the UKB-PPP sub-cohort and the UKB imaging sub-cohort each involved about 10% of the total participants, with different sampling schemes. The UKB-PPP study randomly selected blood samples collected at baseline and its GWAS discovery cohort did not include the participants in the COVID-19 imaging study. Upon matching the individual ID between the UKB-PPP discovery cohort and the UKB participants with the WMH measures, we found 5832 overlapped participants which account for about 16% of the total UKB-PPP discovery cohort and about 10% of the WMH cohort. We expect even smaller overlap between the UKB-PPP participants with other neuroimaging measures as WMH had the lowest missing rate. However, since we were unable to access the individual-level data for the outcome GWAS, we were not able to check the exact overlap between the exposure and outcomes. However, due to the UKB sampling scheme and since the outcome data were meta-analysed from different sources, we expected random and non-substantial overlap between the exposure and outcome datasets which was unlikely to impact our MR results.

GWAS of lacunar stroke

Lacunar stroke has been considered as the common symptomatic presentation of SVD. Hence, we also examined each protein in its association with lacunar stroke besides the neuroimaging outcomes. The GWAS summary statistics for lacunar stroke were obtained from Traylor et al. based on their meta-analysis pooling TOAST-phenotyped and magnetic resonance imaging (MRI) confirmed lacunar stroke cases of European ancestry (N cases = 6,030, N controls = 248,929). The cases were recruited from the multicentre DNA Lacunar Stroke studies 1 and 2 based in the UK and from the International Stroke Genetics Consortium. In the TOAST-phenotyped group, lacunar stroke was defined according to the TOAST criteria, as a clinical lacunar syndrome in the absence of non-lacunar infarction on CT or other causes of stroke except SVD. In the MRI-confirmed group, diagnosis was additionally supported by the MRI evidence showing subcortical infarcts ≤15 mm in diameter. Because MRI confirmation made the diagnosis more accurate than using the TOAST criteria alone, we performed a sensitivity analysis with exclusively MRI-confirmed cases to assess whether heterogeneity in phenotyping affected the results. For the sensitivity analysis, we used GWAS summary statistics from Koohi et al. that updated the Traylor et al. study by using the previous (by May 2021) and the newly recruited (by June 2023) MRI-

confirmed cases in the DNA Lacunar Stroke 1 & 2 studies (N cases = 3,199, N controls = 199,55; both of European ancestry). We henceforth used the term - lacunar stroke (LS) - to refer to all cases identified by either TOAST phenotyping or MRI evidence. The cases diagnosed according to the MRI were specifically referred to as MRI-confirmed LS.

GWAS of systolic blood pressure

High systolic blood pressure (SBP) is a major risk factor for sporadic SVD. To account for its effect in the MR analysis, we obtained GWAS summary statistics for systolic blood pressure (SBP) from Evangelou et al. who conducted a meta-analysis (N=757,601) on SBP harmonizing data from cohorts of European ancestry.

Genetic instruments

Harmonisation was first performed between the GWAS datasets for each protein-outcome pair to find the common SNPs and to ensure consistent effect alleles. SNPs in *cis* association with the proteins (± 1 Mb of the gene-coding region) and below genome-wide significance level ($P < 5 \times 10^{-8}$) were eligible for the instruments. Among them, LD clumping was performed with the PLINK2 software to select uncorrelated SNPs ($r^2 < 0.01$) as the genetic instruments. The 1000 Genome of European ancestry was used as the reference panel. We dropped palindromic SNPs on which we were unable to infer effect alleles confidently. Since the GWAS summary statistics were obtained from different sources and their coverage varied, not all protein-outcome pairs could be offered an instrument that existed in both datasets and satisfied all the above criteria. The missing pattern varied by outcomes: among the 19 missing pairs, 1 protein (IGFBP2) had no instrument when paired with WMH, MD, and FA as the outcomes; 7 and 9 assays were unavailable when paired with lacunar stroke and CMB as the outcomes, respectively.

MR sensitivity analyses

Three assumptions must be satisfied in order to make the estimates from Mendelian Randomization (MR) valid. First, the genetic instruments must be associated with the exposure. Second, the genetic instruments are independent of potential confounders. Third, the genetic instruments can affect the outcome only through its effect on the exposure. To check these assumptions and ensure the validity of our results, we performed four sensitivity analyses.

In the first sensitivity analysis, we changed the instruments to the lead SNPs from each conditionally independent *cis* credible set reported by Sun et al. (cross-referencing their Supplemental Table 16). Specifically, Sun et al. performed sum of single-effects regression (SuSiE) in a conditional analysis framework to fine-map independent genetic signals for each protein. Based on their findings, we used the *cis* SNPs with the highest posterior inclusion probability (PIP) in each independent credible set as the instruments. In the case when the lead SNPs were unavailable in the outcome GWAS, we used the SNPs with the second highest PIP. The beta coefficient, standard error, and *P* values derived from the conditional analysis were used to represent the SNP-protein association.

In the second sensitivity analysis, we made a regional plot for each potentially causal protein using LocusZoom website. Within a window of ± 200kb, we identified the protein-coding genes next to the candidate ones. Of note, five of these neighbouring genes (i.e. *HAVCR1* and *HAVCR2* next to *TIMD4*; *SCGB3A1* next to *FLT4*; *CR1* and *CD34* next to *CD46*) have also been implicated in Alzheimer's disease or other dementias. To rule out the possibility that the neighbouring proteins were causal for SVD, we tested their associations with SVD using the conditionally independent lead SNPs as the instruments.

In the third sensitivity analysis, we conducted an external replication using the overlapped assays on the SomaScan platform with the same MR approach.

In the fourth sensitivity analysis using multivariable MR (MVMR), we derived the uncorrelated SNPs ($r^2 < 0.01$) as the genetic instruments for systolic blood pressure (SBP) using LD clumping (PLINK2 software). To minimize horizontal pleiotropy and to be consistent with our other MR analyses, SNPs within ± 1 Mb of the respective protein's gene-coding region and below genome-wide significance level ($P < 5 \times 10^{-8}$) were eligible as instruments. When the instrument for SBP was unavailable under the $P < 5 \times 10^{-8}$ threshold (as in the cases for METAP1D and for the NPTX1-LS pair), we relaxed the criteria to $P < 1 \times 10^{-5}$. The MVMR (version 0.4) R package was used to estimate the causal effects.

sc-eQTL in human brain cells and PBMCs

We examined whether the gene expression of the candidates had been identified in brain or peripheral blood mononuclear cells based on the findings of Bryois et al. and Yazar et al. We obtained the single-cell gene expression data across 8 brain cells (incl. astrocytes, endothelial

cells, excitatory neurons, inhibitory neurons, microglia, oligodendrocytes, oligodendrocyte precursor cells / committed OPCs), and that across 14 immune-related PBMCs (incl. CD4 naive/central memory T cell, CD4 effector memory/TEMRA, CD4 SOX4 T cell, CD8 naive/central memory T cell, CD8 effector memory, CD8 S100B T cell, naïve/ immature B cell, memory B cell, natural killer cell, natural killer recruiting cell, classical monocytes, non-classical monocytes, dendritic cell, and plasma cell).

For the proteins with gene expression found in these cells, we further queried if their *cis* single-cell expression quantitative trait loci (sc-eQTL) had been identified. Because both reference studies were limited by the lack of power in their sc-eQTLs discovery, they reported sc-eQTLs determined at an FDR threshold of 5% instead of a genome-wide threshold (e.g. $P < 5 \times 10^{-8}$). As this limitation would further cause insufficient power in the colocalization and the full summary statistics covering the genetic region as required by the colocalization test was not available, we decided not to run the formal test. Instead, we checked whether each candidate protein's *cis* sc-eQTL (if detected) was tested in the GWAS of the proteins or the outcomes. If it was, we then examined whether the SNP identified as a *cis* sc-eQTL was also associated with protein abundance or SVD.

Cross-sectional and survival analyses

Cognitive performance

SVD and its related pathologies, including white matter lesions, lacunes, and cerebral microbleeds can impair performance across multiple cognitive domains. Studies have suggested that executive functions and processing speed are the most vulnerable domains to SVD, while other cognitive aspects such as attention, memory, and visuospatial function can also be affected. At the UKB baseline, all participants were invited to complete the reaction time test and the pairs matching test as a measure of information processing speed and visual declarative memory, respectively. Cognitive tests on other domains were also conducted but either within a subset or being introduced later in the imaging subcohort. For our analysis, we used the baseline results of reaction time and pairs matching since almost all participants completed both tests. Specifically, we used the mean response time (milliseconds) for a correct match and the number of incorrect matches in Round 2 as the results for reaction time and pairs matching tests, respectively (UKB data field 20023 and 399).

Stroke and dementia

SVD is a leading contributor to the vascular pathology of dementia. It can also manifest as ischemic stroke or cerebral haemorrhage. To assess whether the proteins causal for SVD can further predict relevant clinical events, we used the algorithmically-defined all-cause stroke and all-cause dementia as the outcomes. Developed by the UK Biobank Outcome Adjudication Group, the algorithm took a predefined ICD code list to document the earliest occurrence of any dementia or stroke based on hospital admission records and death certificates. We excluded prevalent dementia or stroke cases at the study baseline. Since self-report was allowed as a data source only for the cases identified at baseline, this exclusion criterion also ruled out self-reported cases. The predefined ICD codes and their accuracy had been evaluated and reported. We did not investigate subtypes of dementia or stroke because they often have mixed pathology and may share SVD-induced lesions. Moreover, in the case of vascular dementia, the positive predictive value of its diagnostic codes ranged from 19% to 91%, making it unreliable to use.

Competing risks

All-cause deaths other than stroke- or dementia-related were considered as competing risks. We ascertained the causes of death based on the UKB-linked death certificates. A participant was identified as censored due to a competing risk if he or she died during the follow-up without dementia or stroke being recorded as the primary or secondary causes of death.

Covariate categorization

The baseline values of age (years), sex (male/ female), Townsend deprivation index (continuous), BMI (continuous), smoking (Never/ Previous/ Current), alcohol drinking (Never/ Previous/ Current), total cholesterol (continuous), and LDL (continuous) were obtained from the corresponding UKB data fields directly. The categories of *Previous* and *Never* were combined as *Not current* for the variables alcohol drinking and smoking, respectively. Systolic blood pressure (SBP; mmHg) was calculated as the average value between the two automatic readings at baseline. If the automatic readings were unavailable, SBP was calculated as the average value between the two manual readings at baseline. Diabetic status at baseline, including both Type I and II, was ascertained based on the self-reports on touchscreen, verbal interviews, hospital admission records, and whether the glycated haemoglobin (HbA1c) concentration was $\geq$ 48 mmol/mol. An indication from any of the above sources prior to or at the UKB baseline was considered a prevalent case. The

total years of education were calculated based on the self-reported level of quantification. The participants who reported “College or University degree” or “Other professional qualifications” were assigned with 20 years of education; the participants who reported “NVQ or HND or HNC or equivalent” were assigned with 18 years of education; the participants who reported “A levels/AS levels or equivalent” were assigned with 13 years of education; the participants who reported “O levels/GCSEs or equivalent” or “CSEs or equivalent” were assigned with 10 years of education; the participants who answered “none of the above” were considered to have no formal qualifications and were assigned with 7 years of primary education. The participants who preferred not to answer were coded as “not available”.

Regression modelling

In the linear regression models for reaction time, age was fitted as linear and quadratic terms to allow for a curvilinear relationship. In the secondary analysis, besides age and sex, we additionally conditioned on education in years, Townsend deprivation index, BMI, smoking, alcohol drinking, SBP, total cholesterol, LDL, and baseline diabetes. We fitted SBP, total cholesterol, and LDL as linear terms. We modelled age, education in years, Townsend deprivation index, and BMI as both linear and quadratic terms since the likelihood ratio test comparing the models with and without each quadratic term showed significant result ($P<0.05$, two-sided).

In the negative binomial models for matching errors, we modelled age as a linear term in the primary analysis. In its secondary analysis, we additionally conditioned on the same set of covariates as above but fitted all continuous variables as linear terms alone since their quadratic terms did not show significance.

In the Fine-Gray models and the Cox models, all continuous covariates except age were fitted as linear terms in both the primary and secondary analyses. Age at the baseline was fitted as linear and quadratic terms due to the significance of its quadratic term.

Mediation analyses were performed to examine whether the effects of the proteins on dementia or stroke were mediated by high systolic blood pressure. First, a multivariable linear regression was fitted to estimate the effect of each protein’s level on SBP, conditioning on age (linear and quadratic terms) and sex. Then, logistic regression was used to model the

impact of SBP on the risk of dementia or stroke during the follow-up while accounting for each protein's level, age (linear and quadratic terms), and sex. When dementia was the outcome, we additionally adjusted for education and APOE $\varepsilon 4$ carrier status in the mediator and outcome models to be consistent with our primary analyses. The direct and mediated effects were computed using the *mediation* package (version 4.5.0) in R.

Supplemental Figures

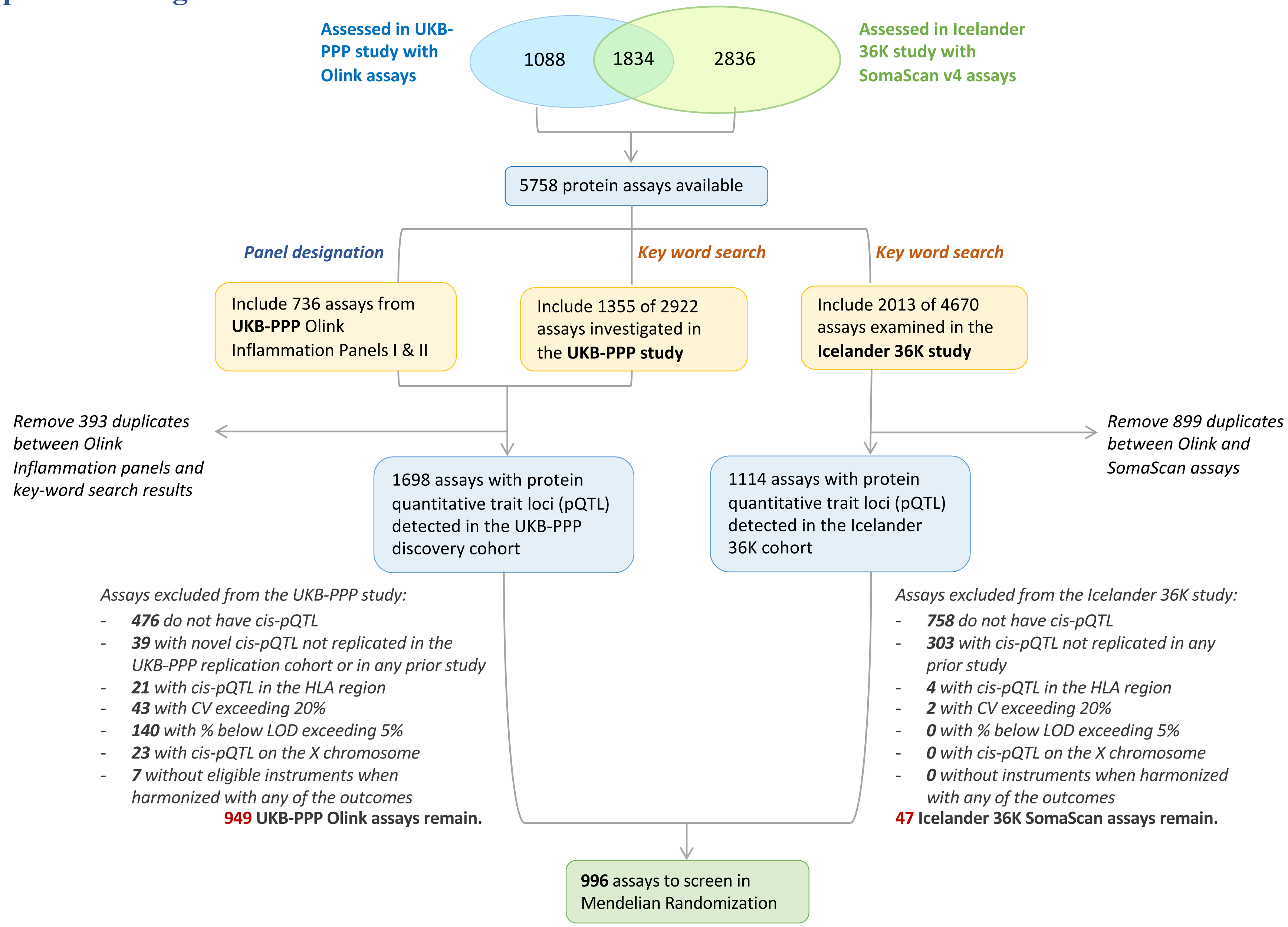

Figure S1. A flowchart showing the selection of circulating proteins.

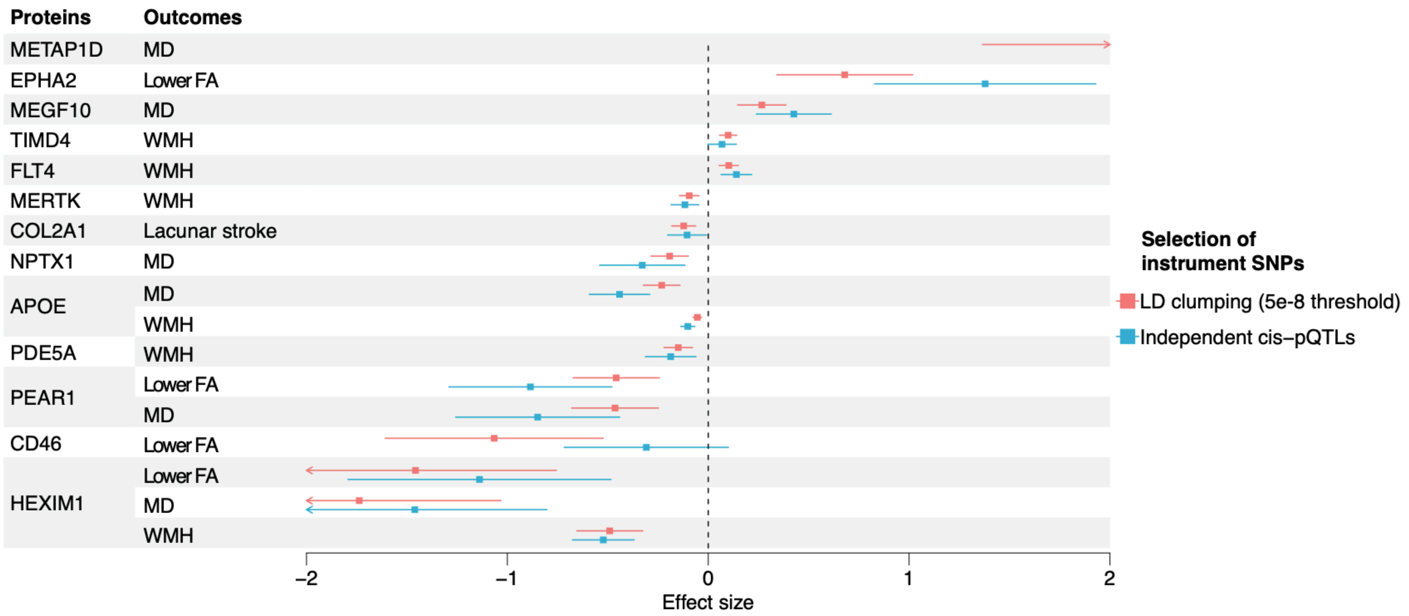

Figure S2. A plot comparing the MR estimates between two methods for instrument selection. In the primary analysis (red colour), the genetic instruments were identified in *cis* association (± 1 Mb) with the protein with a genome-wide *P* threshold at 5×10^{-8}. LD clumping was performed to ensure that the chosen SNPs were uncorrelated. In the sensitivity analysis (blue colour), conditionally independent *cis* pQTLs identified by Sun et al. were used as the instruments. When the *cis* pQTL did not exist in the outcome GWAS, a proxy SNP with the highest posterior inclusion probability in the credible set was used as the instrument. For the METAP1D – MD pair, the instrument SNP identified by LD clumping was the same as the cis pQTL of METAP1D derived from the conditional analysis. Hence, only one MR estimate was shown for this pair.

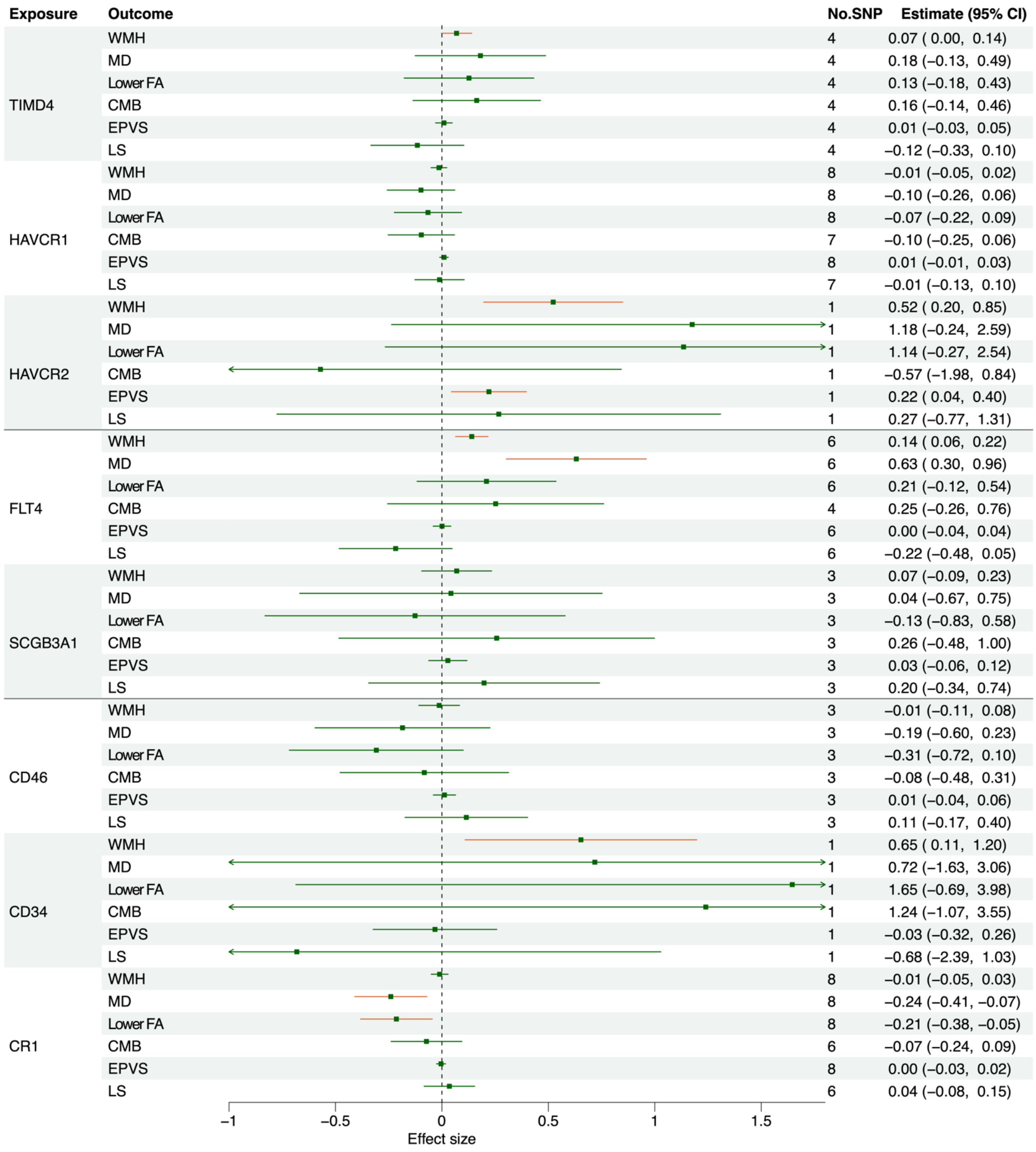

Figure S3. A plot showing the MR estimates of three candidate proteins and their neighbouring proteins. *cis* pQTLs identified by Sun et al. were used as the instrument SNPs to better differentiate the signals coming from different protein-coding genes in one genetic region. Orange line indicates a significant effect with $P<0.05$, two-sided.

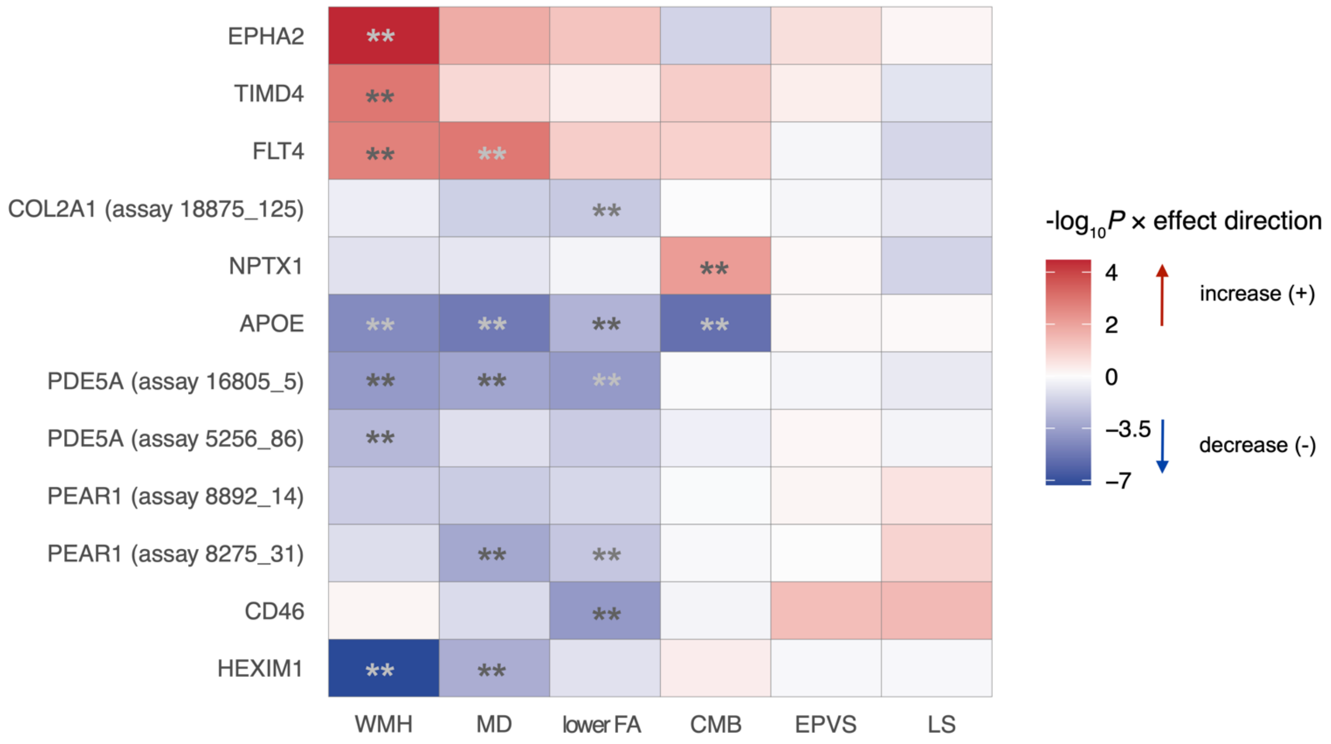

Figure S4. Replication results using summary statistics from the Iceland 36K study. **Red colour** indicates a harmful effect while **blue colour** suggests a protective effect per 1-unit increase in the normalized expression level of circulating protein abundance. The shade of the colour corresponds to *P* values, with darker colour representing stronger evidence for a causal association. False discovery rate (FDR) was calculated via the Benjamin–Hochberg method to account for multiple testing among the 6 outcomes. PEAR1 and PDE5A both had two corresponding assays on the SomaScan platform, and they were all tested. METAP1D and MERTK were not available on the SomaScan platform. MEGF10 assay analysed from the SomaScan platform did not have eligible MR instruments when harmonized with any of the outcome datasets. In total, 72 protein-outcome pairs were tested and accounted for in the FDR correction. At an FDR < 5%, 19 pairs showed a significant association. ** FDR<0.05.

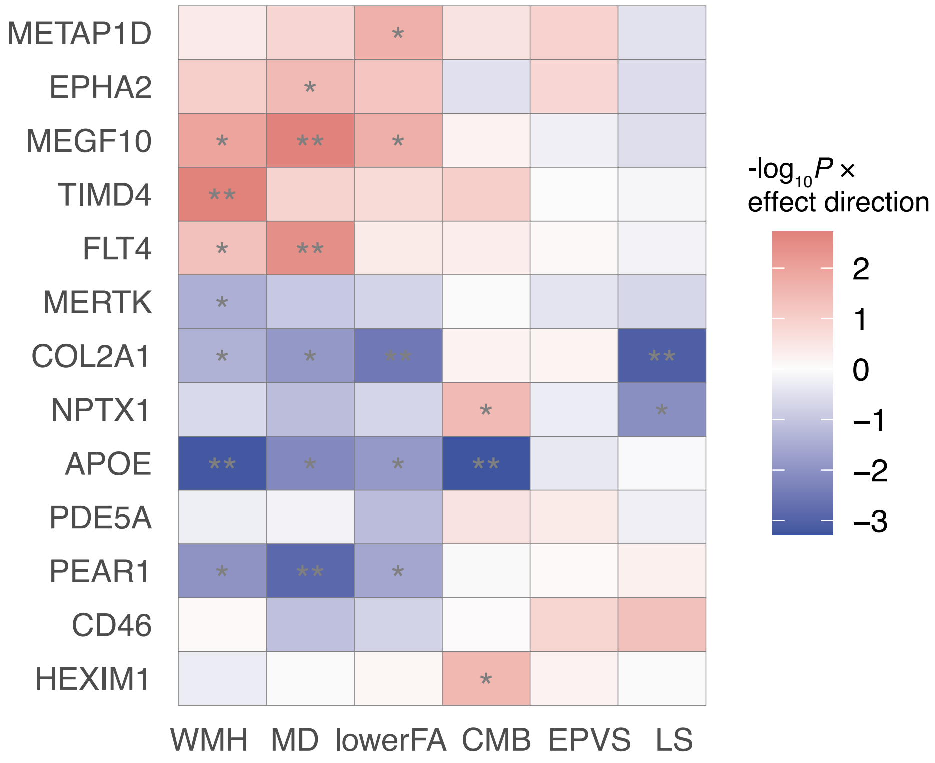

Figure S5. Multivariable MR estimating the effect of individual protein on SVD conditional on SBP. **Red colour** indicates a harmful effect while **blue colour** suggests a protective effect per 1-unit increase in the normalized expression level of circulating protein abundance. The shade of the colour corresponds to *P* values, with darker colour representing stronger evidence for a causal association. False discovery rate (FDR) was calculated via the Benjamin–Hochberg method to account for multiple testing among the 6 outcomes. In total, 78 protein-outcome pairs were tested conditional on SBP and accounted for in the FDR correction. *P<0.05. ** FDR<0.05.

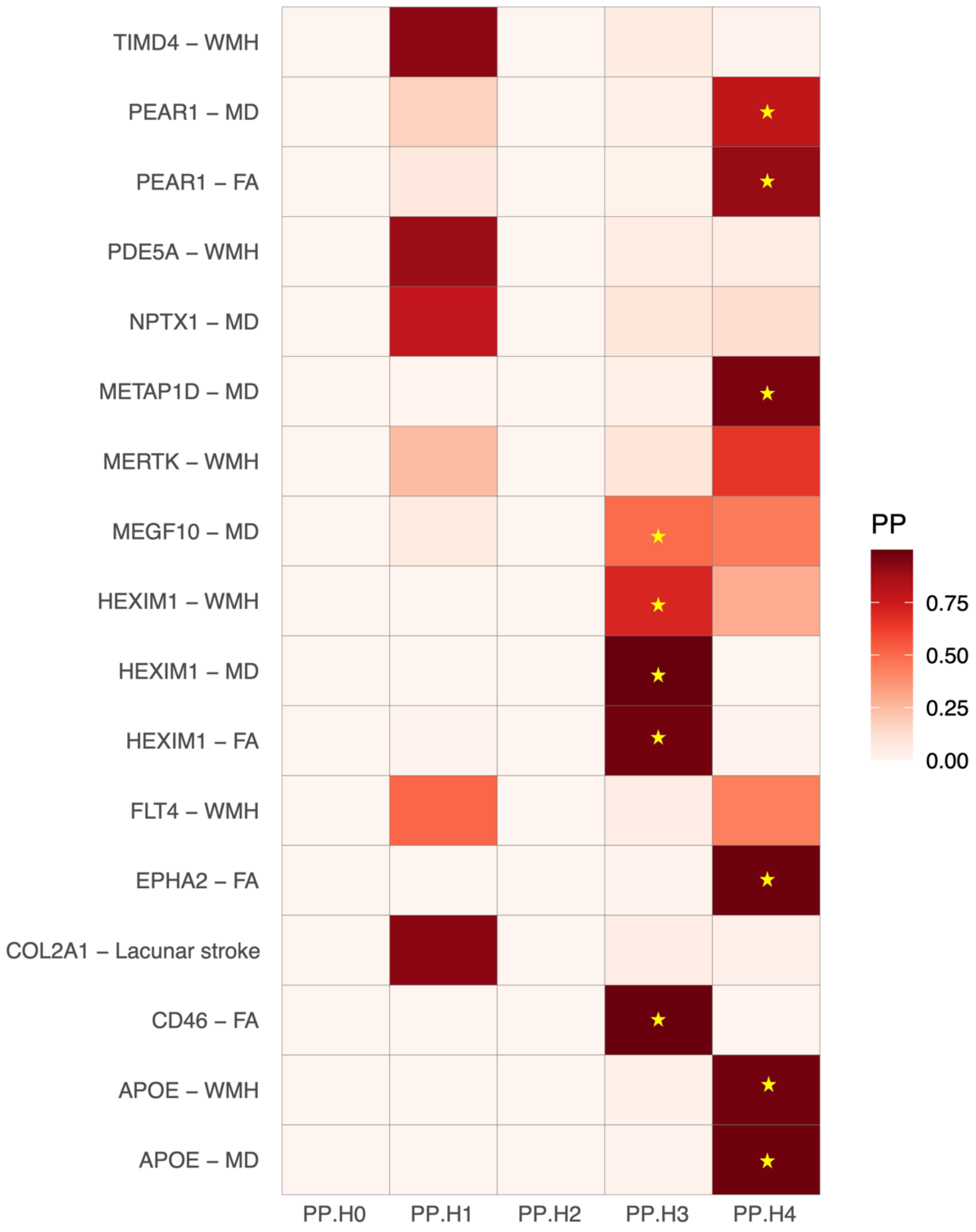

Figure S6. A heatmap of the posterior probability from pairwise colocalization between each of the 17 significant pairs identified by the primary MR. Each column represents the posterior probability (PP) estimated under each hypothesis (H_0-H_4) using the Approximate Bayes Factor (ABF) analysis. The default prior $p_{12} = 1 \times 10^{-5}$ and ±200kb window within the gene-coding region were used in the analysis. Pairs that had either a marginal or conditional colocalization were given a star.

Supplemental Tables

Table S1. Search terms by pathophysiological processes involved in SVD

Pathophysiological processes	Search terms
**Inflammation**	arachi , B cell , basophil , BDNF , caspase , ccl , ccr , CD , ceramid , chemoat , chemokin , chemota , cluster of differentiation , colony-stimulating factor , complemen , cox-1 , cox-2 , CRP , cx , cytokine , dendritic , eosinophil , fingolimod , globulin , granulocyte , growth factor , growth, helper cell , hla , hypoxia-inducible factor , IgA , IgD , IgE , IGF , IgG , IgM , IL- , immunoglobulin , interferon regulatory factor , interferon , interleukin , LCN-2 , leukemia inhibitory factor , leuko , leukotrien , lipocalin , lipoxyge , lps , LTB , lymphocyte , macrophage , mast cell , MCP-1 , MCP , MHC , monocyte chemoattractant protein-1 , monocyte , MPO , myeloperoxidase , natural killer , neutrophil chemoattractant , NGF , NOD2 , OPG , osteoprotegerin , phospholipas , PLVAP , prostacyc , prostaglandin , Ptch , RAGE , retinoic , S1P , shh , sonic hedge , sphingo , sphingosine-1-phosphate receptor , T cell , TGF , th17 cell , thromboxan , TLR , TM4SF , TNF , toll , Transforming growth , treg , trophic , tumor necrosis , VLA
**Endothelial activation and blood-brain barrier dysfunction**	actin , Activated Protein C , adam , adenosine , adhesion , AhR , Akt , albumin , androstane , Ang1 , ap-1 , asct , atp , angio , astrocyte , Bcrp , BDNF , Breast cancer resistance protein , cadherin , catenin , cathepsi , cdh , Cerebral cavernous malformations 2 pr , CFTR , CGN , channel , chymase , CINC , cingulin , clathrin , claudin , Cldn , collagen , connexin , CTNNA , cx , cyclophilin , cytoske , calpain , dag1 , desmo , dystrogl , EBA , elastas , elastin , EP-1 , exchanger , endot*, endothelial nitric oxide synthase , fibroblast, Fibronectin , fbn , FGF , fibrillin , flk , Flk1 , FXR , Gap junction , GDNF , gelatinase , gja , gjb , gjc , glucuronide , glycosamino , glyt , GPIba , GPIb_ , GPIIb , GRO- , gsk , gtp , glycoprote , heparan sul , heparan sulfate , histamin , hmit , hocl , hspg , htra , hyalur , IGF1R , IIIa , integrin , icam- , ICAM , JAM- , jnk , junction , Junctional adhesion , K-dependent protein C , lamin , leukotrien , lipoprotein receptor , lipoprotein , lrp , lxr , mac-1 , macroglobulin , matrix , MCAO , mmp , membrane , monocarboxylate trans , mtx , MYZAP , ndst , nid1 , nidog , nitroprusside , NKeff:%Kir+ , Notch-3 , occludin , ocln , Ornithine aminotransferase , pai-1 , pbc , pcn , pcr , PDGF Rb , PDGF-BB , PDGF , perlecan , PGI , pi-3 , pk , plasma membrane , plasmin , platelet derived , podo , pregnane , prostacyclin , Protein kinase b , protein s100 , proteinase , proteogly , pten , pxr , pericyte , permea , peroxisome proliferators-activated receptor , PPAR , RANTES , riip , robo , s1p , sdc , SDF1 , SELPLG , semaphorin , Serotonin N-acetyltra , sglt , slc , SLC16a1 , sLeptin R , smit , snat , solute carrier , src , syndecan , sele , selectin , sell , selp , TFPI , tissue factor pathway inhibitor , tunica , taut , tetraspa , tgf , tie-2 , Tight junction , TIMP , Tissue-type plasminogen , tps , Transferrin receptor , Transforming growth factor , transport protein , transporter , transporting , trypsin , tryptase , urokinase , vcl , VCAM, vdr , vegf , Vesicle transport , vinculin , Vitamin K-dependent protein S , ZO- , ZO-1 , zonula
**Oxidative stress**	eNOS , integrin , Lp-PLA-2 , NADPH , NFKB , nitric oxi*, nitric oxide synthase , NOX , nuclear factor kappa , oxidase , paraoxonase , peroxidase , SOD , superoxide dismutase

**Neuronal and glial function**	apolipoprotein, cam-, camk , CK-BB , eaat , GFAP , glia , glial fibrillary acidic protein , glut1 , glutamate , MBP , myelin basic protein , Neuron-specific enolase , neuron , neuropilin , NMDA , NR2A , NR2B , NSE , receptor , Sodium-dependent , TRP , TRPV , wnt
**Clotting, vascular remodelling, and metabolic reprogramming**	ACE, adrenomedullin, angiopoietin , angiotensin-converting enzyme , coagulation , collagen , d-dimer , dectin , erythrocyte , fibrinog , fibrinogen , histone acetyltransferase , homocysteine , KDM5 , PAI-1 , PF , PIGF , Placental growth factor , plasminogen activator inhibitor , plasminogen , platelet factor , platelet , PLP , Proteolipid protein , red blood , S100 , SIRT1 , sirtuin , thrombin , thrombo , thrombomodulin , TPA , vWF , Willebrand , zymogen protein C

Table S2. Sources of GWAS summary statistics for SVD-related outcomes

	Outcomes	GWAS catalogue	Sample size	Ancestry	PubMed ID
**Lacunar stroke (LS)**	TOAST-phenotyped or MRI-confirmed LS	GCST90014122	6030 cases, 248929 controls	European	33773637
	MRI-confirmed LS (sensitivity analysis)*	Koohi et al. 2024	3199 cases, 19955 controls	European	38527140
**MRI-imaging features of SVD**	White matter hyperintensity volume (WMH)		55291	European	
	Mean diffusivity (MD)		36460	European	
	Fractional anisotropy (FA)		36533	European	
	Cerebral microbleeds (CMB)	GCST011707	3556 cases, 22306 controls	25106 Europeans, 130 East Asians, 204 South East Asians, 422 African Americans or Afro-Caribbeans	32913026
	Enlarged perivascular space (EPVS) (i.e. extensive white matter perivascular space burden)	GCST90244151	9324 cases, 29274 controls	European	37069360

* *Overlapping with Traylor et al. data but including newly recruited MRI-confirmed lacunar stroke cases*

Table S3. The candidate proteins with their gene expression found in 8 brain cell types

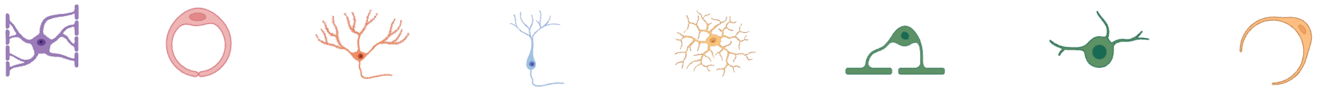

Astrocytes	Endothelial cells	Excitatory neurons	Inhibitory neurons	Microglia	Oligodendrocytes	OPCs / COPs	Pericytes
CD46	PDE5A	**PDE5A**	**PDE5A**	METAP1D	**CD46**	**CD46**	PDE5A
MERTK	CD46	**CD46**	**CD46**	EPHA2	**PDE5A**	PDE5A	CD46
EPHA2	HEXIM1	HEXIM1	METAP1D	CD46	METAP1D	MEGF10	MERTK
PDE5A	FLT4	NPTX1	HEXIM1	TIMD4	MEGF10	METAP1D	FLT4
METAP1D	PEAR1	METAP1D	TIMD4	MERTK			EPHA2
APOE	METAP1D	TIMD4					PEAR1
MEGF10	EPHA2						
	MERTK						

Red bolded: cis eQTLs were identified for PDE5A and CD46 in excitatory neurons, inhibitory neurons, oligodendrocytes, and oligodendrocyte precursor cells (OPCs) / committed OPCs (COPs). Illustrations icons cited from BioRender.

Table S4. The candidate proteins with their gene expression identified in 14 PBMCs

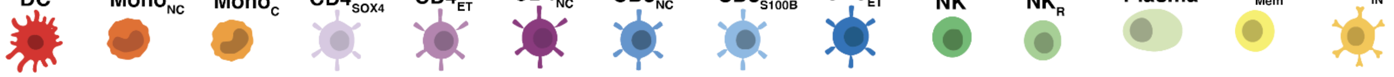

DC	Mono$_{NC}$	Mono$_{C}$	CD4$_{SOX4}$	CD4$_{ET}$	CD4$_{NC}$	CD8$_{NC}$	CD8$_{S100B}$	CD8$_{ET}$	NK	NK$_{R}$	Plasma	B$_{Mem}$	B$_{IN}$
CD46	MERTK	MERTK	CD46	TIMD4	APOE	TIMD4	CD46	TIMD4	TIMD4	CD46	CD46	CD46	CD46
EPHA2	CD46	CD46	HEXIM1	CD46	**TIMD4**	CD46	FLT4	CD46	CD46	HEXIM1	HEXIM1	HEXIM1	HEXIM1
HEXIM1	HEXIM1	HEXIM1	METAP1D	HEXIM1	CD46	FLT4	HEXIM1	FLT4	NPTX1	METAP1D	METAP1D	METAP1D	METAP1D
METAP1D	METAP1D	METAP1D		METAP1D	**FLT4**	PDE5A	METAP1D	PDE5A	PDE5A				
					PDE5A	HEXIM1		HEXIM1	HEXIM1				
					**HEXIM1**	METAP1D		METAP1D	METAP1D				
					**METAP1D**								

Red bolded: cis eQTLs were identified for TIMD4, FLT4, HEXIM1, and METAP1D in CD4 Naive/Central memory T cells. Illustration icons cited from Yazar et al.

Table S5. The SNP-protein and SNP-outcome associations for the reported sc-eQTLs

VarID	cell_type	protein	CHR	BP	A0	A1	sc-eQTL summary statistics		GWAS on proteins	
							BETA.eQTL	P.eQTL	BETA.pQTL	P.pQTL
rs2745979	Excitatory neurons	CD46	1	207844874	G	C	-0.338523	0.000735	0.0362729	1.10E-05
rs4844620	Inhibitory neurons	CD46	1	207807556	G	A	-0.404444	0.00185745	0.0015482	0.86412864
rs2724384	Oligodendrocytes	CD46	1	207756858	A	G	-0.55842	9.54E-10	0.00250013	0.78028993
rs2745979	OPCs / COPs	CD46	1	207844874	G	C	-0.562866	8.08E-10	0.0362729	1.10E-05
rs76820754	CD4 Naive/Central memory T cell	FLT4	5	180509737	G	A	0.16280114	2.91E-07	0.0834538	9.37E-16
rs4632173	CD4 Naive/Central memory T cell	HEXIM1	17	45183416	A	G	0.15532623	1.00E-06	-0.0584748	4.32E-15
rs116843903	CD4 Naive/Central memory T cell	HEXIM1	17	45716674	G	A	-0.1342742	2.43E-05	0.00558682	0.70227951
rs55667037	CD4 Naive/Central memory T cell	METAP1D	2	172054834	A	G	-0.1489128	2.78E-06	0.0193875	0.26986776
rs10930517	CD4 Naive/Central memory T cell	METAP1D	2	172149028	G	T	0.11564912	0.00028161	0.0256714	0.00078433
rs6829903	Excitatory neurons	PDE5A	4	119585729	C	T	-0.23265	2.45E-10	0.13085	6.21E-57
rs7673476	Inhibitory neurons	PDE5A	4	119327528	G	A	-0.198052	7.74E-05	0.125192	5.39E-57
rs10013652	Oligodendrocytes	PDE5A	4	119371101	A	G	-0.15726	0.00319566	0.128723	1.13E-58
rs72805105	CD4 Naive/Central memory T cell	TIMD4	5	156870905	A	C	0.12766693	6.02E-05	-0.0720444	1.38E-10

Table S5 Continued (aligned with the upper table by VarID).

VarID	GWAS on WMH		GWAS on MD		GWAS on FA		GWAS on PVS burden		GWAS on LS		GWAS on CMB	
	BETA.WMH	P.WMH	BETA.MD	P.MD	BETA.FA	P.FA	BETA.EPVS	P.EPVS	BETA.LS	P.LS	BETA.CMB	P.CMB
rs2745979	-0.0035346	0.63003547	-0.0292869	0.3532946	-0.0424954	0.17568634	0.0038	0.2252	0.015	0.5132	-0.024	0.4281
rs4844620	-0.000892	0.91214441	-0.0816988	0.01877198	-0.11108	0.00131535	0.0029	0.4204	0.022	0.3778	-0.0211	0.5378
rs2724384	0.00020934	0.97913873	-0.0759438	0.02742142	-0.104255	0.00232391	0.0026	0.3692	0.0217	0.3668	-0.0153	0.6461
rs2745979	-0.0035346	0.63003547	-0.0292869	0.3532946	-0.0424954	0.17568634	0.0038	0.2252	0.015	0.5132	-0.024	0.4281
rs76820754	0.0116174	0.26505134	-0.0365669	0.41687994	-0.0355245	0.42685758	-0.001	0.2934	-0.0134	0.6961	0.0797	0.0697
rs4632173	0.0384228	1.19E-08	0.113026	9.55E-05	0.0677927	0.01852636	-0.0027	0.06398	0.0094	0.6615	-0.0321	0.2702
rs116843903	-0.0204655	0.11960188	-0.0753381	0.18161428	-0.136322	0.01513422	-0.0014	0.7023	-0.0602	0.209	NA	NA
rs55667037	-0.003481	0.82120976	-0.0809614	0.22124273	-0.0051902	0.93711524	0.0046	0.478	-0.0428	0.3928	0.0682	0.3359
rs10930517	-0.004744	0.48299985	0.0100664	0.72895211	0.0429995	0.13698594	0.0014	0.8746	0.0093	0.6694	0.0236	0.4138
rs6829903	-0.0107743	0.1380575	-0.0370613	0.23488268	-0.0551385	0.0757042	0.0018	0.665	-0.0306	0.1775	0.0135	0.6514
rs7673476	-0.0165697	0.01723614	-0.0139476	0.64069303	-0.041803	0.15963238	0.003	0.8198	-0.0187	0.3989	0.004	0.8895

rs10013652	-0.0137178	0.05156678	-0.0361379	0.23272016	-0.0585623	0.0518215	0.0038	0.8036	-0.012	0.5899	1.00E-04	0.9969
rs72805105	0.0199826	0.05896985	-0.0460518	0.31455095	-0.0130568	0.77362946	-0.0058	0.5008	0.0289	0.3996	-2.00E-04	0.9969

Table S6. Baseline characteristics of the UKB-PPP cohort by incident dementia or stroke status

	Non-dementia (N=46,343)	Dementia (N=1,228)	Non-stroke (N=46,303)	Stroke (N=1,268)	Overall (N=47,571)
**Age at baseline (years)**					
Mean (SD)	56.5 (8.17)	64.7 (4.97)	56.6 (8.20)	61.4 (6.72)	56.7 (8.20)
**Sex (n %)**					
Female	25228 (54.4%)	621 (50.6%)	25290 (54.6%)	559 (44.1%)	25849 (54.3%)
Male	21115 (45.6%)	607 (49.4%)	21013 (45.4%)	709 (55.9%)	21722 (45.7%)
**Education (years)**					
Mean (SD)	14.8 (5.34)	13.2 (5.64)	14.8 (5.34)	13.8 (5.64)	14.8 (5.35)
**Townsend deprivation index**					
Mean (SD)	-1.26 (3.14)	-1.13 (3.23)	-1.27 (3.13)	-0.877 (3.24)	-1.26 (3.14)
**BMI**					
Mean (SD)	27.4 (4.75)	27.6 (4.87)	27.4 (4.75)	28.1 (4.92)	27.4 (4.75)
**Smoking (n %)**					
Current	4855 (10.5%)	105 (8.6%)	4742 (10.2%)	218 (17.2%)	4960 (10.4%)
Not current	41488 (89.5%)	1123 (91.4%)	41561 (89.8%)	1050 (82.8%)	42611 (89.6%)
**Alcohol drinking (n %)**					
Current	42489 (91.7%)	1053 (85.7%)	42402 (91.6%)	1140 (89.9%)	43542 (91.5%)
Not current	3854 (8.3%)	175 (14.3%)	3901 (8.4%)	128 (10.1%)	4029 (8.5%)
**Systolic blood pressure (mmHg)**					
Mean (SD)	138 (18.6)	145 (19.2)	138 (18.6)	145 (20.2)	138 (18.6)
**Total cholesterol (mmol/L)**					
Mean (SD)	5.68 (1.15)	5.52 (1.25)	5.68 (1.15)	5.51 (1.23)	5.68 (1.15)
**Low-density lipoprotein (mmol/L)**					
Mean (SD)	3.55 (0.874)	3.42 (0.928)	3.55 (0.874)	3.44 (0.920)	3.54 (0.875)
**Diabetes (n %)**					
No	43634 (94.2%)	1060 (86.3%)	43604 (94.2%)	1090 (86.0%)	44694 (94.0%)
Yes	2709 (5.8%)	168 (13.7%)	2699 (5.8%)	178 (14.0%)	2877 (6.0%)
**APOE ε4 carrier (n %)**					
No	34743 (75.0%)	895 (72.9%)	34673 (74.9%)	965 (76.1%)	35638 (74.9%)
Yes	11600 (25.0%)	333 (27.1%)	11630 (25.1%)	303 (23.9%)	11933 (25.1%)
**Follow-up till dementia/ stroke/ censoring (years)**					
Mean (SD)	14.2 (2.29)	9.22 (3.28)	14.2 (2.28)	8.19 (3.90)	14.0 (2.64)*
**Reaction Time (ms)**					
Median [25th% - 75th%]	551 [491-625]	594 [528-688]	551 [491-626]	567 [508-656]	551 [492-628]
**Errors in pairs matching**					
Median [25th% - 75th%]	3 [2-6]	4 [2-7]	3 [2-6]	4 [2-6]	3 [2-6]

*years of follow-up from baseline to incident dementia, stroke, or censoring, whichever occurred first

Appendix. The list of 996 proteins

A1BG, ACHE, ACP5, ACVRL1, ACYP1, ADA, ADA2, ADAM15, ADAM22, ADAM23, ADAM8, ADAM9, ADAMTS13, ADAMTS15, ADAMTS16, ADAMTS5, ADAMTS8, ADAMTSL2, ADAMTSL4, ADAMTSL5, ADD1, ADGRB3, ADGRD1, ADGRE1, ADGRE2, ADGRE5, ADGRG1, ADH1B, ADIPOQ, ADM, AFM, AGRN, AGRP, AGT, AHCY, AHSG, ALCAM, ALDH3A1, ALPP, AMBN, AMBP, AMY1A_AMY1B_AMY1C, ANG, ANGPT1, ANGPT2, ANGPTL1, ANGPTL3, ANGPTL4, ANGPTL7, ANKMY2, ANXA11, AOC3, AP1G2, APBB1IP, APCS, APMAP, APOA1, APOA4, APOBR, APOC1, APOD, APOE, APOF, APOH, APOL1, APPL2, ARHGAP25, ARHGAP45, ASAH1, ASAH2, ASGR1, ASGR2, ATOX1, ATRAID, ATRN, AXL

B3GNT7, B3GNT8, B4GALT1, BANK1, BCAM, BCHE, BCR, BID, BMP10, BMPER, BOC, BPI, BSG, BST2, BTC, BTD

C1QA, C1QC, C1QL2, C1QTNF1, C1QTNF3, C1QTNF5, C1QTNF9, C1R, C1RL, C1S, C3, C7, C8B, CACYBP, CAPG, CASP3, CASP7, CASP8, CAT, CBLIF, CCDC126, CCDC50, CCDC80, CCER2, CCL11,
CCL13, CCL14, CCL15, CCL16, CCL17, CCL19, CCL2, CCL20, CCL21, CCL22, CCL23, CCL24, CCL25, CCL3, CCL4, CCL5, CCL7, CCL8, CCN2, CCS, CD101, CD109, CD14, CD160, CD163, CD164, CD177, CD1C, CD200, CD200R1, CD207, CD209, CD22, CD226, CD244, CD248, CD27, CD274, CD276, CD28, CD2AP, CD300A, CD300C, CD300E, CD300LF, CD300LG, CD302, CD33, CD34, CD36, CD38, CD4, CD40, CD46, CD48, CD5, CD55, CD58, CD59, CD5L, CD6, CD69, CD7, CD70, CD72, CD74, CD79B, CD80, CD83, CD84, CD86, CD8A, CDA, CDCP1, CDH15, CDH17, CDH2, CDH3, CDH5, CDH6, CDHR1, CDHR2, CDHR5, CDKN1A, CDNF, CDON, CEACAM1, CEACAM16, CEACAM19, CEACAM21, CEACAM6, CEACAM8, CELA2A, CELA3A, CELSR2, CFD, CFH, CFHR2, CFHR4, CFHR5, CFI, CGREF1, CHAD, CHCHD10, CHL1, CKAP4, CKMT1A_CKMT1B, CLEC3B, CLEC4A, CLEC4C, CLEC4D, CLEC4G, CLEC7A, CLIP2, CLMP, CLPS, CLSTN2, CLU, CNTFR, CNTN1, CNTN2, CNTN3, CNTN4, CNTN5, CNTNAP2, COL15A1, COL18A1, COL1A1, COL2A1, COL3A1, COL4A1, COL5A1, COL6A3, COL9A1, COLEC12, COMP, CPA4, CPB2, CR1, CR2, CRELD1, CRELD2, CRHBP, CRIM1, CRISP3, CRISPLD2, CSF1, CSF1R, CSF3, CSF3R, CSPG4, CST1, CST7, CTBS, CTHRC1, CTRB1, CTRB2, CTRC, CTRL, CTSB, CTSC, CTSD, CTSE, CTSF, CTSH, CTSL, CTSO, CTSS, CTSV, CTSZ, CX3CL1, CXADR, CXCL1, CXCL10, CXCL11, CXCL12, CXCL13, CXCL16, CXCL17, CXCL5, CXCL6, CXCL9, CYTL1

DAG1, DAPP1, DARS1, DBH, DBNL, DCBLD2, DCXR, DDAH1, DDHD2, DDX58, DKK1, DKK3, DKK4, DKKL1, DNAJB6, DNER, DNMBP, DNPH1, DPP10, DSC2, DSG2, DSG3, DTD1, DTX3

EBAG9, EBI3_IL27, ECE1, ECM1, EDAR, EDN1, EFEMP1, EGF, EGFL7, EGFR, EGLN1, EIF2AK2, ELN, ENAH, ENPP5, ENPP7, EPHA1, EPHA2, EPHA4, EPHB4, EPHB6, EPO, EPPK1, EPS8L2, ERBB2, ERBB3, ERBB4, ERMAP, ESAM, ESM1, EVI5

F10, F11, F12, F13B, F2R, F5, F7, FABP1, FABP9, FAH, FAS, FASLG, FCAMR, FCAR, FCER1A, FCER2, FCGR2A, FCGR2B, FCGR3B, FCN1, FCRL1, FCRL2, FCRL4, FCRL5, FCRL6, FCRLB, FGA, FGF21, FGF23, FGF5, FGFBP1, FGFBP2, FGFBP3, FGFR2,

FGFR4, FGG, FGL1, FHIT, FIS1, FLRT2, FLT4, FN1, FNDC1, FOLR1, FOLR2, FOLR3, FOXO1, FST, FSTL3, FTCD, FUOM, FUT3_FUT5

GAL, GALNT10, GALNT16, GALNT2, GALNT3, GALNT7, GAS6, GC, GCHFR, GCNT1, GDF15, GDNF, GFRA1, GFRA2, GFRA3, GFRAL, GGACT, GHR, GIMAP7, GMFG, GMPR, GMPR2, GNPDA2, GP1BA, GP2, GP5, GP6, GPA33, GPHA2, GPNMB, GPR37, GSN, GSR, GZMA, GZMB

HAVCR1, HAVCR2, HCLS1, HDGF, HEG1, HEXIM1, HGF, HGFAC, HIP1R, HMCN2, HNMT, HPCAL1, HPGDS, HRG, HS3ST3B1, HSPG2, HYAL1

ICAM1, ICAM2, ICAM3, ICAM4, ICAM5, ICOSLG, IFI30, IFNAR1, IFNGR1, IFNGR2, IFNLR1, IGDCC4, IGF1R, IGF2R, IGFBP2, IGFBP3, IGFBP6, IGFBP7, IGFBPL1, IGFLR1, IGLL1, IGSF21, IGSF3, IGSF8, IL10RB, IL12B, IL16, IL17C, IL17D, IL17RA, IL17RB, IL18, IL18BP, IL18R1, IL19, IL1R1, IL1R2, IL1RAP, IL1RL1, IL1RL2, IL1RN, IL22, IL27RA, IL2RA, IL32, IL4R, IL5RA, IL6R, IL6ST, IL7, IL7R, INHBB, IQGAP2, IRAK4, ISG15, ISLR2, ISM1, ITGA11, ITGA2, ITGA6, ITGAL, ITGAM, ITGAV, ITGB2, ITGB5, ITGB6, ITGB7, ITGBL1, ITIH3, ITIH4, ITPR1

JAM2, JAM3

KDR, KEL, KIR2DL2, KIR2DL3, KIR2DL4, KITLG, KLK7, KLKB1, KLRB1, KLRD1, KLRF1, KLRK1, KRT19, KYNU

LAG3, LAIR1, LAIR2, LAMA4, LAMB1, LAMC2, LAMP3, LCAT, LCN15, LCN2, LECT2, LEG1, LEPR, LGALS4, LGALS9, LGMN, LHPP, LIFR, LILRA2, LILRA3, LILRA5, LILRA6, LILRB1, LILRB2, LILRB4, LILRB5, LIPF, LPL, LPO, LRG1, LRIG1, LRP11, LRPAP1, LRRN1, LRTM2, LSP1, LTBP2, LTBP3, LTBR, LY6D, LY75, LY9, LY96, LYVE1, LZTFL1

MAMDC4, MANEA, MANF, MAPK9, MARCO, MATN2, MBL2, MCAM, MCEMP1, MCFD2, MDH1, MEGF10, MEGF9, MENT, MEPE, MERTK, MET, METAP1D, MFAP4, MGLL, MGMT, MGP, MIA, MIF, MILR1, MMP1, MMP10, MMP12, MMP3, MMP7, MMP8, MMP9, MOCS2, MPO, MRC1, MSR1, MST1, MSTN, MVK, MXRA7, MXRA8, MYDGF, MYOM3, MZB1

NAA80, NAAA, NAGK, NAGPA, NBL1, NCAM1, NCAM2, NCF2, NCR1, NCR3LG1, NCS1, NELL2, NFASC, NFATC1, NFKB1, NFKBIE, NHLRC3, NID1, NID2, NME3, NOS1, NPC2, NPL, NPTX1, NPTX2, NPTXR, NRCAM, NRP1, NRP2, NT5C3A, NTF3, NTRK2, NTRK3, NUB1, NUMB, NXPH3

OCLN, OGFR, OLFM4, OLR1, OMD, OMG, ORM1, OSCAR, OSM, OSMR

PAG1, PARP1, PAXX, PCBD1, PCDH1, PCDH12, PCDH17, PCDH7, PCDH9, PCDHB15, PCOLCE, PCYOX1, PDCD1, PDCD1LG2, PDCD5, PDCD6, PDE5A, PDGFA, PDGFC, PDGFRA, PDGFRB, PEAR1, PEBP1, PENK, PEPD, PF4, PFKFB2, PGA4, PGF, PGLYRP2, PHACTR2, PHYKPL, PI16, PIGR, PIK3AP1, PIK3IP1, PILRA, PILRB, PINLYP, PKD1, PKD2, PKLR, PLA2G10, PLA2G15, PLA2G2A, PLA2G4A, PLA2G7, PLA2R1, PLAT, PLAU, PLAUR, PLEKHA7, PLG, PLXNA4, PNLIPRP2, PODXL, PODXL2, PON1, PON3, PPCDC, PPL, PRDX2, PRDX5, PRELP, PRG2, PRG3, PRKG1,

PROC, PROK1, PROS1, PRRT3, PRSS2, PRSS22, PRSS8, PRTN3, PSG1, PSG3, PSG4, PSG5, PSMD9, PSMG4, PSTPIP2, PTGDS, PTGFRN, PTGR1, PTH1R, PTPRB, PTPRC, PTPRF, PTPRH, PTPRM, PTPRN2, PTPRR, PTPRS, PTPRZ1, PTX3, PVALB, PVR

QSOX1, QSOX2

RAB11FIP3, RABEP1, RANBP1, RARRES2, REG4, RELT, RET, RHOC, RIDA, RLN2, RNASE1, RNASE4, RNASE6, RNF149, ROBO1, ROBO2, ROBO4, ROR1, RTN4IP1, RTN4R

S100A11, S100A12, S100A13, S100A4, S100P, SAMD9L, SAT2, SCAMP3, SCARA5, SCARB2, SCARF1, SCARF2, SCG3, SCGB1A1, SCGB3A1, SCGB3A2, SCLY, SCN4B, SCRN1, SDC1, SDC4, SDCCAG8, SDK2, SEC31A, SECTM1, SELE, SELENOP, SELL, SELP, SELPLG, SEMA3C, SEMA3E, SEMA3F, SEMA4D, SEMA5A, SEMA7A, SERPINA3, SERPINA4, SERPINA5, SERPINA6, SERPINB1, SERPINB8, SERPINC1, SERPIND1, SERPINE1, SERPINF1, SERPINF2, SERPING1, SERPINI1, SFRP4, SHBG, SHMT1, SIAE, SIGLEC1, SIGLEC10, SIRPA, SIRPB1, SIRT2, SLA2, SLAMF7, SLC39A14, SLC39A5, SLC9A3R1, SLITRK1, SLMAP, SMOC2, SMPD1, SMPDL3A, SNED1, SNX15, SOD2, SOD3, SORCS2, SPAG1, SPINK2, SPINK4, SPINT2, SPON1, SPRY2, SSC4D, SSC5D, SSNA1, ST13, ST3GAL1, STAMBP, STAT2, STX7, STX8, SUOX, SUSD5, SVEP1

TACC3, TAFA5, TAPBPL, TBCA, TCN1, TCN2, TEK, TF, TFF2, TFPI, TFPI2, TFRC, TGFA, TGFB1, TGFBI, TGFBR3, TGOLN2, THBD, THBS2, THBS4, THPO, THTPA, THY1, TIE1, TIMD4, TIMP2, TIMP4, TINAGL1, TJAP1, TLR3, TMEM106A, TMEM190, TMEM25, TMPRSS11D, TMPRSS5, TNFAIP2, TNFAIP6, TNFAIP8L2, TNFRSF10A, TNFRSF10B, TNFRSF10C, TNFRSF11A, TNFRSF11B, TNFRSF12A, TNFRSF13B, TNFRSF13C, TNFRSF14, TNFRSF17, TNFRSF19, TNFRSF1A, TNFRSF1B, TNFRSF21, TNFRSF4, TNFRSF6B, TNFRSF8, TNFRSF9, TNFSF10, TNFSF11, TNFSF12, TNFSF13, TNFSF13B, TNFSF14, TNFSF8, TOP2B, TP53I3, TPK1, TPP1, TPSAB1, TRAF2, TREM1, TREM2, TRIM21, TRIM25, TRIM5, TRIM58, TSPAN8, TTR, TXN, TYRO3

UCMA, UNC5C, UROD

VAMP8, VAT1, VCAM1, VEGFA, VEGFB, VEGFC, VMO1, VNN1, VSIG2, VSIR, VTI1A, VWA1, VWC2L

WARS, WFIKKN1, WFIKKN2, WIF1, WISP1, WISP2, WNT9A, WWP2

XCL1
